# Edge-rich molybdenum disulfide tailors carbon-chain growth for selective hydrogenation of carbon monoxide to higher alcohols

**DOI:** 10.1038/s41467-023-42325-z

**Published:** 2023-10-26

**Authors:** Jingting Hu, Zeyu Wei, Yunlong Zhang, Rui Huang, Mingchao Zhang, Kang Cheng, Qinghong Zhang, Yutai Qi, Yanan Li, Jun Mao, Junfa Zhu, Lihui Wu, Wu Wen, Shengsheng Yu, Yang Pan, Jiuzhong Yang, Xiangjun Wei, Luozhen Jiang, Rui Si, Liang Yu, Ye Wang, Dehui Deng

**Affiliations:** 1 State Key Laboratory of Catalysis, Collaborative Innovation Center of Chemistry for Energy Materials, Dalian Institute of Chemical Physics, Chinese Academy of Sciences, Dalian, 116023 China; 2 State Key Laboratory of Physical Chemistry of Solid Surfaces, Collaborative Innovation Center of Chemistry for Energy Materials, College of Chemistry and Chemical Engineering, Xiamen University, Xiamen, 361005 China; 3 University of Chinese Academy of Sciences, Beijing, 100049 China; 4 National Synchrotron Radiation Laboratory, University of Science and Technology of China, Hefei, 230029 China; 5 Shanghai Synchrotron Radiation Facility, Shanghai Advanced Research Institute, Chinese Academy of Sciences, Shanghai, 201204 China; 6 Shanghai Institute of Applied Physics, Chinese Academy of Sciences, Shanghai, 201204 China

**Keywords:** Two-dimensional materials, Heterogeneous catalysis, Materials for energy and catalysis

## Abstract

Selective hydrogenation of carbon monoxide (CO) to higher alcohols (C_2+_OH) is a promising non-petroleum route for producing high-value chemicals, in which precise regulations of both C-O cleavage and C-C coupling are highly essential but remain great challenges. Herein, we report that highly selective CO hydrogenation to C_2-4_OH is achieved over a potassium-modified edge-rich molybdenum disulfide (MoS_2_) catalyst, which delivers a high CO conversion of 17% with a superior C_2-4_OH selectivity of 45.2% in hydrogenated products at 240 °C and 50 bar, outperforming previously reported non-noble metal-based catalysts under similar conditions. By regulating the relative abundance of edge to basal plane, C_2-4_OH to methanol selectivity ratio can be overturned from 0.4 to 2.2. Mechanistic studies reveal that sulfur vacancies at MoS_2_ edges boost carbon-chain growth by facilitating not only C-O cleavage but also C-C coupling, while potassium promotes the desorption of alcohols via electrostatic interaction with hydroxyls, thereby enabling preferential formation of C_2-4_OH.

## Abstract (web summary)

Controllable synthesis of higher alcohols from CO hydrogenation remains a great challenge. Here the authors show that potassium-decorated edge-rich MoS_2_ enables selective CO hydrogenation to higher alcohols by regulating the reaction kinetics of C-O cleavage, C-C coupling and desorption of alcohols.

## Introduction

CO hydrogenation provides an attractive non-petroleum route for producing higher alcohols (C_2+_OH), which are widely used as chemical feedstocks, fuels, fuel additives, and solvents, etc.^1–4^. In this reaction, formation of C_2+_OH typically involves two critical steps, i.e. (i) C-O cleavage of adsorbed CO (CO*) or alkoxyls (CH_*x*_O*, *x* = 1−4) to generate adsorbed C (C*) or alkyls (CH_x_*, *x* = 1 − 3) species and (ii) C-C coupling among the C*, CH_x_*, CO*, or CH_*x*_O* for carbon-chain growth^5^. In these steps, C-O cleavage as the foregoing step for C-C coupling is of essential significance but also inevitably causes decomposition of the generated alcohols. Such a seesaw effect makes the selectivity of C_2+_OH hardly controllable.

A variety of catalysts have been explored for CO hydrogenation to C_2+_OH, such as Rh-based catalysts^6,7^, modified Cu-based methanol-synthesis catalysts^8–10^, modified Fischer-Tropsch-synthesis (FT-synthesis) catalysts^11–14^, and Mo-based catalysts^15–19^. Among these catalysts, the Rh-based catalysts are beneficial for ethanol synthesis but suffer from high price. The modified Cu-based methanol-synthesis catalysts usually give rise to a large proportion of methanol among alcohol products^8–10^, since the C-C coupling for producing higher alcohols over the catalyst typically undergoes a sluggish aldol-type condensation mechanism. Modified FT-synthesis catalysts such as Co-based and Fe-based catalysts, exhibit high activity in CO hydrogenation because Co and Fe are classical active metals for C-O dissociation and C-C coupling^20^. But the irrepressible C-O cleavage and the random C-C coupling on the open surface of FT catalysts typically lead to wide distribution of carbon number in alcohols^12,13^. In addition, the above three catalysts usually suffer poor stability caused by phase separation, sintering, or sulfur poisoning under working conditions, which hinders their industrial application^6,21–25^. The Mo-based catalysts are well known in sulfur tolerance^21^, but have relatively lower activity for C-O dissociation and C-C coupling, thus usually leading to suppressed carbon-chain growth with a high methanol selectivity and typically requiring high reaction temperatures (>300 °C) and harsh pressures (>80 bar) for the production of C_2+_OH^15,17–19,26–28^. Though great progresses have been made for the development of catalysts for higher alcohols synthesis in previous works, the limited CO conversion (<10%) or C_2-4_OH selectivity (<40%) under mild reaction conditions is still insufficient and need to be improved. The development of highly efficient non-noble metal-based catalysts for the CO hydrogenation to C_2+_OH under mild conditions, requires precise regulations of active site in a nano-level to control both the C-O cleavage and C-C coupling, which are the keys for realizing controllable carbon-chain growth and selective formation of specific C_2+_OH but remain great challenges.

Herein, we report that on the basis of a nano channel-confined growth mechanism, uniform nano-arrays of potassium-modified edge-rich MoS_2_ (ER-MoS_2_-K) is prepared for highly selective CO hydrogenation to C_2-4_OH. At relatively low temperature of 240 °C and low pressure of 50 bar, a high CO conversion of 17% is reached with a superior C_2-4_OH selectivity of 45.2% in hydrogenated products, surpassing that of previously reported non-noble metal-based catalysts under similar conditions. By reducing the lateral size of MoS_2_ to enrich the edges for boosting the carbon-chain growth, the C_2-4_OH to methanol selectivity ratio is overturned from 0.4 to 2.2, and the selectivity of C_2-4_OH is almost 100% in the C_2+_OH products. In-situ characterizations combined with theoretical calculations reveal the sulfur vacancies (SVs) at the edge of the ER-MoS_2_-K as the active sites for the CO hydrogenation to C_2-4_OH, where not only the C-O cleavage of CH_*x*_O* is facilitated for the generation of CH_x_* intermediate, but also the subsequent C-C coupling between CH_x_* and CO* is more favored for the growth of carbon-chain than deep hydrogenation to methane. The potassium promoter promotes the desorption of alcohols via electrostatic interaction with the hydroxyl of alcohols, thereby improving the selectivity toward C_2-4_OH.

## Results

### Structure and performance of edge-rich MoS_2_

**Fig. 1:**
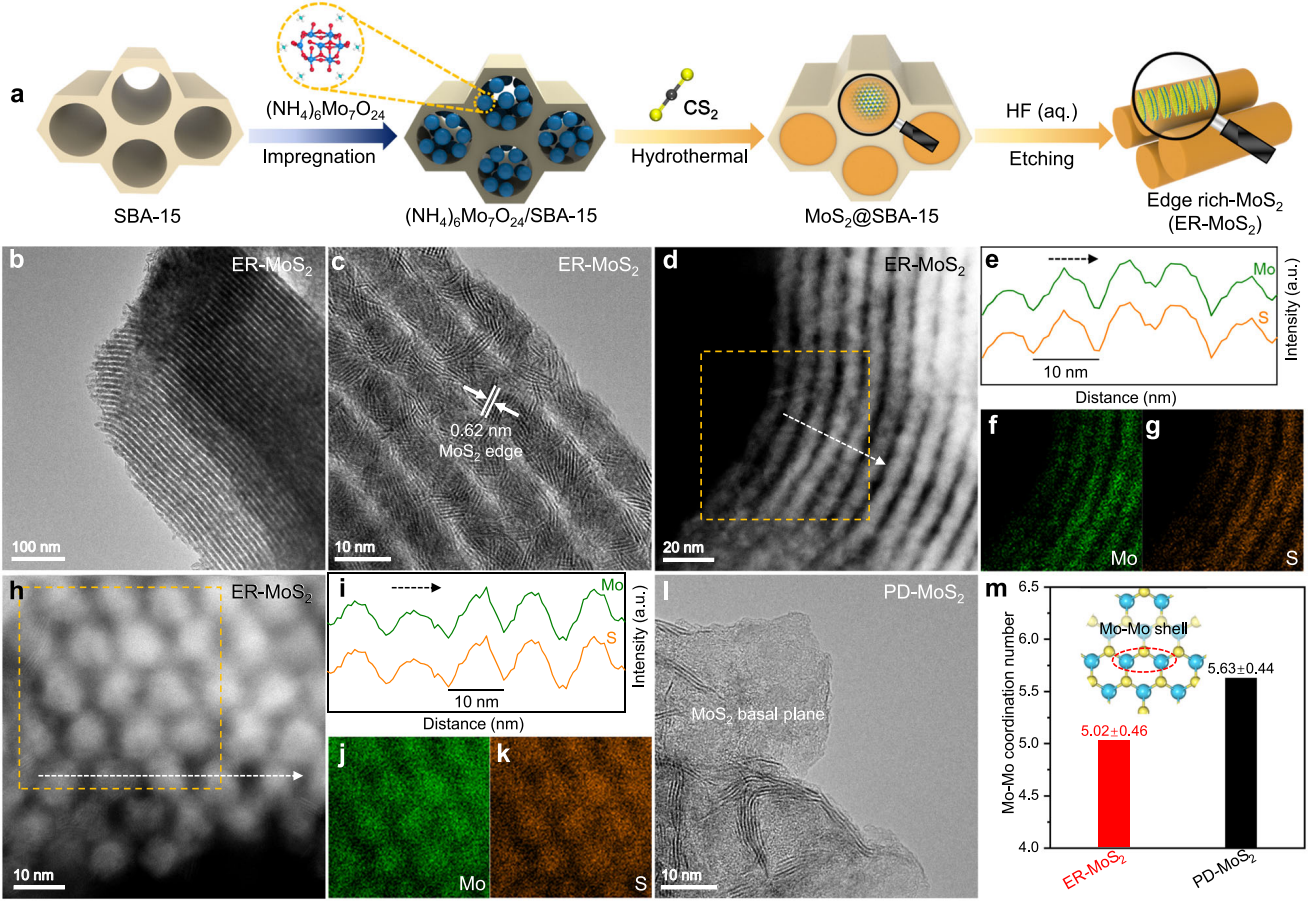
Structural characterization of different MoS_2_. **a** Schematic illustration of the synthesis of ER-MoS_2_. **b**, **c** Transmission electron microscopy (TEM) images of ER-MoS_2_. **d**–**k** High-angle annular dark field scanning transmission electron microscopy (HAADF-STEM) images (**d**, **h** side view and top view, respectively), EDX line scans (**e**, **i**), and EDX mappings (**f**, **g**, **j**, **k**) of ER-MoS_2_. **l**, TEM image of PD-MoS_2_. **m** The coordination number of the Mo-Mo shell for ER-MoS_2_ and PD-MoS_2_.

Edge-rich MoS_2_ (denoted as ER-MoS_2_) was synthesized on the basis of nano channel-confined growth mechanism by using SBA-15 (a kind of ordered mesoporous silica) as a hard template to restrict the lateral growth of MoS_2_ in the confined space of its mesoporous channels (Fig. 1a, Supplementary Fig. 1, and Supplementary Table 1). After removing the SBA-15 template, structural characterizations show that the obtained ER-MoS_2_ consists of nanosheets with small lateral sizes and abundant edges assembled in nano-array morphology with uniform linear channels as derived from the template (Fig. 1b-k, Supplementary Fig. 2a). As shown in energy-dispersive X-ray (EDX) line scans of ER-MoS_2_ (Fig. 1e, i), the diameter of an array is around 10 nm, corresponding to the pore diameter of SBA-15 (Supplementary Fig. 1). Basal plane-dominated MoS_2_ (denoted as PD-MoS_2_) with relatively large lateral sizes was also synthesized as a reference catalyst to investigate the roles of MoS_2_ edge and basal plane in the CO hydrogenation reaction (Supplementary Fig. 2b). X-ray diffraction (XRD) patterns demonstrate that both ER-MoS_2_ and PD-MoS_2_ exhibit typical characteristics of hexagonal 2H-MoS_2_ crystal (Supplementary Fig. 2c). The PD-MoS_2_ with large lateral sizes mainly exposes the basal plane of MoS_2_ sheets (Fig. 1l, Supplementary Fig. 2b), in sharp contrast with the ER-MoS_2_ which mainly exposes edges. This is also reflected by the Fourier-transformed extended X-ray absorption fine structure (EXAFS) spectra, which show that the ER-MoS_2_ has less Mo-Mo coordination than the PD-MoS_2_ (Fig. 1m and Supplementary Fig. 2d, e), further confirming that the ER-MoS_2_ possesses more edge sites than the PD-MoS_2_^29,30^.

**Fig. 2:**
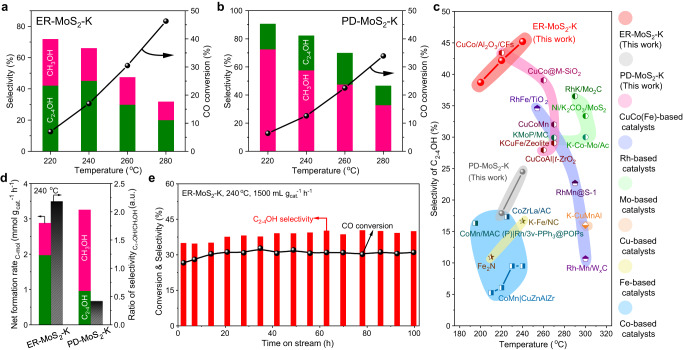
Catalytic performances of different MoS_2_-based catalysts. **a**, **b** Catalytic performances of ER-MoS_2_-K (**a**) and PD-MoS_2_-K (**b**) at 3000 mL g_cat__._^−1^ h^−1^. **c** Comparison in the selectivity of C_2-4_OH over ER-MoS_2_-K, PD-MoS_2_-K and other catalysts reported in literatures (see Supplementary Table 3 for more details). **d** Net formation rate and distribution of alcohol products over ER-MoS_2_-K and PD-MoS_2_-K at 240 °C, 3000 mL g_cat._^−1^ h^−1^. **e** Stability test of the ER-MoS_2_-K catalyst in CO hydrogenation reaction at 1500 mL g_cat__._^−1^ h^−1^. The product selectivity was calculated on a CO_2_-free basis. Catalysts were pretreated in-situ by H_2_ at 300 °C for 1 h before reaction. Reaction activity tests were performed using a tubular fixed-bed reactor at 50 bar and a H_2_/CO of 2.

Potassium was further introduced as a promoter for MoS_2_ but without affecting the overall pore size distributions and structures of MoS_2_ by using an impregnant method with potassium carbonate as the source of potassium (Supplementary Fig. 3, Supplementary Table 2). Catalytic performances of the potassium-modified MoS_2_ (denoted as MoS_2_-K) catalysts for CO hydrogenation were evaluated in a fixed-bed reactor (Fig. 2a, b). Over the ER-MoS_2_-K catalyst with an optimized K/Mo mole ratio of 0.2 (Supplementary Fig. 4), C_2-4_OH are always the primary alcohol product at different temperatures from 220 to 280 °C (Fig. 2a). At relatively low temperature of 240 °C and low pressure of 50 bar with a CO conversion of 17%, a high C_2-4_OH selectivity of 45.2% can be achieved over the catalyst, which is higher than those of previously reported non-noble metal-based catalysts under similar conditions (Fig. 2c, Supplementary Table 3). Moreover, a high yield of 8.41% for C_2-4_OH can also be achieved at 240 °C and 50 bar, while similar yield can only be reached at temperatures above 300 °C and pressures above 80 bar over previously reported MoS_2_-based catalysts (Supplementary Fig. 5, Supplementary Table 4). Interestingly, the distribution of carbon number in the alcohol products is quite narrow and the selectivity of C_2-4_OH in C_2+_OH is almost 100%, indicating the well-controlled carbon-chain growth over the ER-MoS_2_-K catalyst compared with classical Co-based and Fe-based catalysts^12,28^. In contrast to the ER-MoS_2_-K catalyst, methanol becomes the main alcohol product over the PD-MoS_2_-K catalyst with abundant basal planes, especially at lower reaction temperatures (Fig. 2b), and the C_2-4_OH selectivity can only reach 24.5% at best at 240 °C. The formation rate of C_2-4_OH (calculated on carbon mol basis) over the PD-MoS_2_-K catalyst is only 0.96 mmol g_cat__._^−1^ h^−1^ compared with that of 2.0 mmol g_cat__._^−1^ h^−1^ over the ER-MoS_2_-K catalyst. Consequently, the C_2-4_OH to methanol selectivity ratio is overturned from 0.4 to 2.2 by reducing the lateral size of MoS_2_ to enrich the edges (Fig. 2d), thus indicating the important role of MoS_2_ edges in promoting the CO hydrogenation to C_2-4_OH.

The ER-MoS_2_-K catalyst exhibits a high stability with well-maintained CO conversion and C_2-4_OH selectivity in the performance test for 100 h (Fig. 2e). TEM and X-ray absorption spectroscopy (XAS) characterizations show no obvious change in the structure of the ER-MoS_2_-K catalyst after the 100 h of on-stream reaction test (Supplementary Fig. 3a, b, and d). To further investigate the long-term durability of the ER-MoS_2_-K catalyst, the catalytic performances for CO hydrogenation reaction with time-on-stream (TOS) of ~1000 h were also evaluated. Despite desulfurization of MoS_2_ as a common problem causing activity loss is unavoidable^31^, the catalytic performance of ER-MoS_2_-K can be sustained for TOS of 1075 h via regularly replenishing sulfur at every TOS of 300 h by using H_2_S/H_2_ treatment of the catalyst (Supplementary Fig. 6). XRD and HAADF-STEM characterizations show the well-maintained 2H-MoS_2_ crystal phase and channel structure of the used ER-MoS_2_-K catalyst after the long-term reaction test (Supplementary Figs. 6c and 7). These results reveal the ER-MoS_2_-K as a potential catalyst for industrial application combined with regular sulfur maintenance.

To exclude the influence of different morphologies of the ER-MoS_2_-K and PD-MoS_2_-K on their catalytic performances and also to further verify the effect of MoS_2_ edge in improving the selectivity of C_2-4_OH, we prepared another two K-promoted MoS_2_ catalysts possessing similar foam-like morphologies, but consisting of MoS_2_ nanosheets with different structural features, i.e. edge-rich nanosheets and basal plane-dominated nanosheets, which are denoted as ER-MoS_2_^foam^-K and PD-MoS_2_^foam^-K, respectively. These two catalysts were synthesized by using SiO_2_ sphere as a hard template but with different sulfur precursors (see Methods part for detailed synthesis process, Supplementary Fig. 8). XRD patterns demonstrate that the foam-like MoS_2_ samples exhibit typical characteristics of hexagonal 2H-MoS_2_ crystal (Supplementary Fig. 8i). The almost identical Mo K-edge XAS spectra and XPS spectra of different MoS_2_ samples demonstrate that the chemical states of Mo and S in the foam-like samples (ER-MoS_2_^foam^ and PD-MoS_2_^foam^) are basically the same with those in the ER-MoS_2_ and PD-MoS_2_ (Supplementary Fig. 9). In addition, NH_3_ temperature-programmed desorption (NH_3_-TPD) experiment^32^ of all catalysts show similar desorption peaks at ~130 °C corresponding to NH_3_ desorption from the coordinatively unsaturated Mo sites at sulfur vacancies as weak Lewis acid sites (Supplementary Fig. 10), suggesting that the effect of acidity of different catalysts on their performances is neglectable. These results indicate that the foam-like catalysts are reliable references to exclude the morphology effect.

Structural characterizations confirm that both ER-MoS_2_^foam^-K and PD-MoS_2_^foam^-K possess uniform porous frameworks with similar surface areas and pore sizes (Supplementary Fig. 8a, b, d, e, and Supplementary Table 1, Entry 8, 10), yet are different in the lateral size of the MoS_2_ nanosheets (Supplementary Fig. 8c, f). The ER-MoS_2_^foam^-K is featured by small-sized nanosheets with abundant edges (Supplementary Fig. 8e, f), while the PD-MoS_2_^foam^-K mainly exposes large basal planes of MoS_2_ as shown in the TEM images (Supplementary Fig. 8b, c). The edge-length statistical analysis based on the TEM images also demonstrates the markedly smaller lateral size of the ER-MoS_2_^foam^-K than that of the PD-MoS_2_^foam^-K (9 nm *vs*. 741 nm, Supplementary Fig. 8g, h). Interestingly, reaction performance tests show that an obviously higher C_2-4_OH to methanol selectivity ratio of 1.4 is obtained over the ER-MoS_2_^foam^-K compared with that of 0.6 over the PD-MoS_2_^foam^-K (Supplementary Fig. 8j, Supplementary Table 5), which is consistent with the comparison between the ER-MoS_2_-K and PD-MoS_2_-K (Fig. 2d, Supplementary Table 5). These results further confirm the key role of MoS_2_ edges in catalyzing CO hydrogenation to C_2-4_OH.

### Mechanistic understanding of higher alcohols synthesis over MoS_2_

To further understand the catalytic function of MoS_2_ edges in CO hydrogenation, the distribution ratio of CH_*x*_ and CH_*x*_O parts (n_CH*x*_/n_CH*x*O_) in the non-CO_2_ products is analyzed and used as an indicator for the C-O bond dissociation activity of the MoS_2_ catalysts (Supplementary Fig. 11), since CH_*x*_ comes from the dissociated CH_*x*_O. For CO hydrogenation over the ER-MoS_2_-K at 240 °C, the obtained hydrogenated products present a n_CH*x*_/n_CH*x*O_ of 1.45, which is remarkably higher than that of 0.44 over the basal plane-dominated PD-MoS_2_-K catalyst. These results show that C-O cleavage is more favorable over the ER-MoS_2_-K compared with that over the PD-MoS_2_-K, thus indicating that the edge sites of MoS_2_ can facilitate the C-O dissociation of CH_*x*_O to produce CH_*x*_ intermediate.

**Fig. 3:**
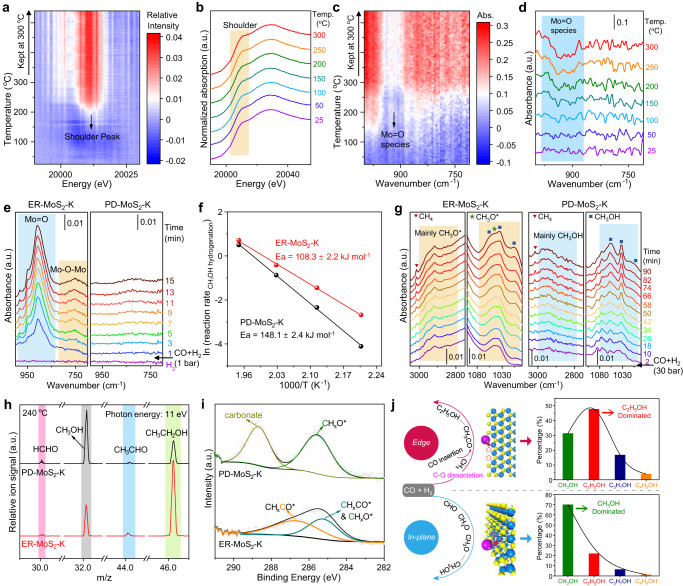
C-O dissociation ability and CO hydrogenation mechanism of different MoS_2_ catalysts. **a**–**d** In-situ ED-XAS (**a**, **b**) coupling in-situ DRIFTS (**c**, **d**) characterizations of ER-MoS_2_-K during H_2_ pretreatment measured with a hyphenated technology. Relative intensity in (**a**) was obtained by subtracting each spectrum to the first spectrum. **e** In-situ DRIFT spectra of CO hydrogenation over the H_2_-pretreated catalysts at 25 °C, H_2_/CO of 2 and 1 bar. **f** Arrhenius plots calculated based on the reaction rate for methanol hydrogenation over PD-MoS_2_-K and ER-MoS_2_-K. **g** In-situ DRIFT spectra of CO hydrogenation over the H_2_-pretreated catalysts at 240 °C, H_2_/CO of 2 and 30 bar. **h** In-situ SVUV-PIMS detection of the CO hydrogenation intermediates and products over the H_2_-pretreated catalysts at 240 °C, H_2_/CO of 2 and 5 bar. **i** Quasi in-situ SRPES detection of the CO hydrogenation intermediates. SRPES spectra were obtained after the treatment of the H_2_-treated catalysts with reaction gas (H_2_/CO = 2) at 240 °C and 5 bar for 0.5 h. **j** Proposed reaction mechanism for CO hydrogenation to methanol and ethanol on edge and in-plane sites of MoS_2_.

To gain deep insights into the active sites at the MoS_2_ edges, the dynamic change in electronic states and evolution of surface oxygen species during the H_2_ reduction pretreatment and subsequent reaction test of the catalyst were monitored by using a hyphenated technology of in-situ time-resolved energy-dispersive X-ray absorption spectroscopy (ED-XAS) and in-situ diffuse reflectance infrared Fourier transform spectroscopy (DRIFTS) characterizations (Supplementary Fig. 12a). The in-situ ED-XAS results show that the absorption edge gradually shifts to higher energies together with an increase in the intensity of its shoulder peak at around 20010 eV during the H_2_ pretreatment process of both ER-MoS_2_-K and PD-MoS_2_-K catalysts, indicating the decrease of the Mo oxidation state with the exposure of coordinatively unsaturated Mo atoms (Fig. 3a, b and Supplementary Fig. 12b, c)^30,33^, which can be ascribed to the formation of sulfur vacancies due to the removal of surface O atoms and some S atoms from MoS_2_ by the H_2_ pretreatment. This is also reflected by the directly detected signals of H_2_O, H_2_S, and SO_2_ during the reduction process in the in-situ mass spectrum for both catalysts (Supplementary Fig. 13). Electron paramagnetic resonance (EPR) characterizations show the rise of a signal at *g* = 2.00, further confirming the existence of SVs on both the reduced ER-MoS_2_-K and PD-MoS_2_-K catalysts (Supplementary Fig. 14a)^34,35^. Moreover, both catalysts possess similar densities of SVs as reflected by their similar integrated intensities of EPR peaks (Supplementary Fig. 14b) and also similar adsorption capacities of NO (89.1 *vs*. 103.3 umol_NO_ g_cat__._^−1^) (Supplementary Fig. 14c). Performance of ER-MoS_2_-K is substantially improved after the H_2_ pretreatment, suggesting the key role of SVs in catalyzing CO hydrogenation (Supplementary Fig. 15).

The removal of surface oxygen is also evidenced by in-situ DRIFTS characterizations (hyphenated with in-situ ED-XAS) of the process, displaying the decreased intensity of the absorption peaks for the vibrations of Mo=O and Mo-O-Mo at around 930 cm^−1^ and 760 cm^−1^, respectively (Fig. 3c, d and Supplementary Fig. 12d, e), which can be attributed to the removal of oxygen adsorbed at the edge and in-plane SVs, respectively^30,36^. It’s worth noting that only the removal of Mo=O species was obviously observed during H_2_ pretreatment of the ER-MoS_2_-K catalyst (Fig. 3c, d), suggesting that the ER-MoS_2_-K catalyst is enriched with edge SVs. In contrast, the PD-MoS_2_-K catalyst possesses more in-plane SVs as reflected by the removal of mainly Mo-O-Mo species during H_2_ pretreatment (Supplementary Fig. 12d, e). In-situ O_2_-adsorption DRIFTS characterizations of the K-free MoS_2_ samples show the rise of mainly Mo=O species on ER-MoS_2_ while mainly Mo-O-Mo species on PD-MoS_2_, also suggesting the totally different SVs distributions on the two kinds of catalysts (Supplementary Fig. 16).

After the in-situ H_2_ pretreatment of the catalysts for the generation of SVs, reaction tests were then performed by using CO/H_2_ mixture or Ar-bubbled CH_3_OH as the feed gas, respectively. In both cases, in-situ DRIFTS characterizations clearly show the reappearance of the Mo-O peaks over the H_2_-pretreated ER-MoS_2_-K catalyst (Fig. 3e, Supplementary Fig. 17), which can be attributed to the formation of Mo-O species from the C-O dissociation of CH_*x*_O at the SVs sites. In comparison, only negligible Mo-O peaks with much weaker intensities were detected over the H_2_-pretreated PD-MoS_2_-K catalyst either with syngas or methanol as the feed gas (Fig. 3e and Supplementary Fig. 17). In addition, the hydrogenation of CH_3_OH to methane reaction was also tested over the two catalysts, in which the ER-MoS_2_-K exhibits a higher activity with a lower activation energy compared with the PD-MoS_2_-K based on the Arrhenius plots (Fig. 3f). These results demonstrate that the SVs at the MoS_2_ edge possess a higher activity for the C-O dissociation than those in the basal plane.

To obtain mechanistic understanding of the CO hydrogenation reaction process on MoS_2_-based catalysts, a series of in-situ characterizations were conducted to capture the reaction intermediates. In-situ high-pressure DRIFTS characterizations show the gradual appearance of surface CH_3_O* species and CH_3_OH on both ER-MoS_2_-K and PD-MoS_2_-K when CO/H_2_ mixture passes through the catalysts at 240 °C (Fig. 3g), where the vibrational bands at 2957, 2916, 2855 and 1044 cm^−1^ can be attributed to CH_3_O* species, while the complicated vibrational bands from 2800 ~ 3000 cm^−1^ and bands at 1056, 1032, 1012 cm^−1^ correspond to the C-H and C-O stretch of CH_3_OH, respectively^37^. It is noteworthy that formation of CH_3_OH was observed as dominating species on the PD-MoS_2_-K catalyst in contrast to the dominating CH_3_O* on the ER-MoS_2_-K (Fig. 3g)^38^. This is supported by our density functional theory (DFT) calculations that the hydrogenation of CH_3_O* to CH_3_OH* is exergonic by 0.81 eV with a low reaction barrier of 0.38 eV over the in-plane SVs but is endergonic by 0.6 eV with a higher barrier of 0.96 eV over the Mo-edge SVs (Supplementary Fig. 18), thus indicating the more favorable formation of CH_3_OH on the PD-MoS_2_-K with rich basal planes than that on the ER-MoS_2_-K with rich edges under the same reaction condition (Fig. 3g). These results explain the higher selectivity toward CH_3_OH in CO hydrogenation over the PD-MoS_2_-K catalyst. As the H_2_-pretreated catalysts were exposed to 5 bar of reaction gas (H_2_/CO = 2) at 240 °C, signals of HCHO, CH_3_OH, CH_3_CHO, and CH_3_CH_2_OH were directly detected by using in-situ synchrotron-based VUV photoionization mass spectrometry (SVUV-PIMS) (Fig. 3h). HCHO and CH_3_CHO could be reaction intermediates for the generation of CH_3_OH and CH_3_CH_2_OH. The intensity ratio of CH_3_OH/CH_3_CH_2_OH follows the trend as mentioned above (Fig. 2a, b) that the main product is CH_3_OH for the PD-MoS_2_-K catalyst and CH_3_CH_2_OH for the ER-MoS_2_-K catalyst.

Quasi in-situ synchrotron radiation photoelectron spectroscopy (SRPES) measurements were conducted to further detect the surface adsorbed species on catalysts (Fig. 3i). Before the measurements, the reduced ER-MoS_2_-K and PD-MoS_2_-K catalysts were treated with the reaction gas (H_2_/CO = 2/1, 5 bar) at 240 °C for 0.5 h. At the binding energy region of C 1 s, two peaks at 285.3 eV and 286.7 eV were detected on the ER-MoS_2_-K catalyst, which can be assigned to the CH_*x*_O* and CH_*x*_CO* species^39^. In contrast, only CH_*x*_O* and carbonate species corresponding to the peaks at 285.6 eV and 288.8 eV were detected without C-C coupled CH_*x*_CO* species on the PD-MoS_2_-K catalyst. Based on the above results, we propose that the hydrogenation of CO to CH_3_CH_2_OH undergoes the following steps,

i. the adsorption and hydrogenation of CO to CH_3_O* (or CH_3_OH*),
ii. the dissociation of CH_3_O* (or CH_3_OH*) to CH_3_*,
iii. the insertion of CO* into CH_3_* to produce CH_3_CO*,
iv. the hydrogenation of CH_3_CO* to CH_3_CHO* and then CH_3_CH_2_OH*,
v. the desorption of CH_3_CH_2_OH*.

The edge SVs of MoS_2_ favor more the synthesis of C_2+_OH by facilitating the dissociation of C-O bond of CH_*x*_O* and then the carbon-chain growth via C-C coupling (Fig. 3j).

### Key factors for tailoring carbon-chain growth

**Fig. 4:**
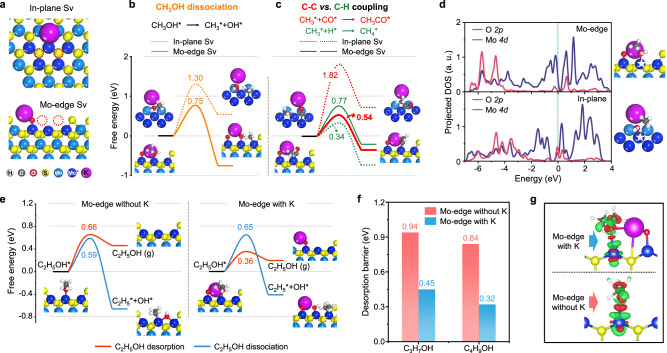
DFT studies of C-O cleavage, C-C coupling, and the desorption of alcohols on double-Sv of MoS_2_. **a** Models of potassium-modified in-plane SVs and Mo-edge SVs. **b** DFT calculated reaction barrier for the dissociation of CH_3_OH on Mo-edge SVs and in-plane SVs. **c** Reaction barrier of the hydrogenation of CH_3_* and the insertion of CO into CH_3_* on Mo-edge SVs and in-plane SVs. **d** Projected DOS of Mo 4*d* orbits and O 2*p* orbits of transition states of methanol dissociation on Mo-edge SVs (up) and in-plane SVs (down). **e** DFT calculations on C_2_H_5_OH* desorption or dissociation on Mo-edge SVs without or with the decoration of potassium. **f** DFT calculated reaction barriers for the desorption of C_3_H_7_OH and C_4_H_9_OH on Mo-edge SVs with or without the decoration of potassium. **g** Side view of differential charge density of the adsorbed C_2_H_5_OH on Mo atoms in Mo-edge SVs with or without the decoration of potassium. The red and green regions indicate electron accumulation and depletion, respectively.

The C-O dissociation and C-C coupling are the key steps for controlling the carbon-chain growth in CO hydrogenation. To gain an atomic level understanding of the higher C_2+_OH selectivity over the edge SVs than that over the in-plane SVs, the activity of the two types of SVs for carbon-chain growth were studied by using DFT calculations. The dissociation of CH_3_OH* and coupling of C_*x*_H_2*x*+1_* (x = 1−3) with CO* or H* were adopted as probing reactions for investigating the C-O dissociation and C-C coupling activities, since these intermediates are common surface species during the reaction. A double-Sv at the Mo-edge or in the basal plane was simulated as the active site (Supplementary Fig. 19a, d). The potassium promoter was introduced into the model by forming a Mo-O-K structure in the adjacent of the double-Sv (Fig. 4a and Supplementary Fig. 19b, c, e, f). This is based on in-situ DRIFTS characterizations of the H_2_ reduction process, which show that the Mo=O species of the ER-MoS_2_ can almost be completely removed by H_2_, while a certain amount of Mo=O species of the ER-MoS_2_-K remains stable even at 300 °C under the decoration of potassium (Supplementary Fig. 20), indicating the formation of Mo-O-K structure with a higher stability than the normal Mo=O.

The dissociation of CH_3_OH* to CH_3_* and OH* over the Mo-edge SVs requires an activation energy of only 0.75 eV, which is notably lower than that of 1.30 eV over the in-plane SVs (Fig. 4b). This indicates that the Mo-edge SVs possess a higher activity for the dissociation of C-O bond to form CH_3_ species, which is essential for the subsequent C-C coupling steps. Analyses of projected density of states (PDOS) reveal the formation of a stronger Mo-O bond in the transition state for CH_3_OH dissociation over the Mo-edge SVs compared with that over the in-plane SVs, as indicated by the lower energy levels of the overlapped part of Mo 4*d* and O 2*p* orbits in the transition states of the former case (Fig. 4d). This leads to lowered C-O dissociation barrier on the Mo-edge SVs.

The reaction energetics of the C_*x*_H_2*x*+1_*-CO* (C-C) coupling and C_*x*_H_2*x*+1_*-H* (C-H) coupling was then investigated on the in-plane and Mo-edge SVs. On the in-plane SVs, the CH_3_*-CO* coupling requires a much higher activation energy of 1.82 eV than the CH_3_*-H* coupling with only a low barrier of 0.34 eV (Fig. 4c). However, on the Mo-edge SVs, the barrier of CH_3_*-CO* coupling (0.54 eV) becomes notably lower than that of CH_3_*-H* coupling (0.77 eV), and similar results was also found in the cases of C_2_H_5_* (0.42 eV for C-C coupling vs 0.76 eV for C-H coupling) and C_3_H_7_* (0.53 eV for C-C coupling vs 0.81 eV for C-H coupling) (Supplementary Fig. 21). In addition, the formation of C_*x*_H_2*x*+1_CO* from C-C coupling on the Mo-edge SVs is also more exergonic in free energy than the formation of C_*x*_H_2*x*+2_ from C-H coupling (Supplementary Fig. 21). These results demonstrate that the C-C coupling between C_*x*_H_2*x*+1_* and CO* for carbon-chain growth is thermodynamically and kinetically more favorable than the C-H coupling between C_*x*_H_2*x*+1_* and H* for alkane formation on the edge SVs of MoS_2_.

Potassium modification plays a key role in improving the selectivity toward alcohols in CO hydrogenation over MoS_2_-based catalysts (Supplementary Fig. 22 and 23). With the decoration of potassium, the selectivity of alcohols significantly increases from <1.5% over the ER-MoS_2_ catalyst to >60% over the ER-MoS_2_-K catalyst at 240 °C (Supplementary Fig. 22a). The above analysis shows that the Mo-edge SVs are highly active for the dissociation of C-O bond, which may lead to decomposition of the formed alcohols. Thus, the timely desorption of the formed alcohols to avoid undesired C-O cleavage is critical for the production of alcohols. To understand the effect of potassium in promoting the production of alcohols, we further conducted DFT calculations for the desorption and C-O cleavage of CH_3_OH and C_2_H_5_OH on Mo-edge SVs with or without the decoration of potassium (Fig. 4e and Supplementary Fig. 24). In the case without the modification of potassium, the C-O cleavage of CH_3_OH* and C_2_H_5_OH* requires activation energies of 0.73 eV and 0.59 eV, respectively, which are lower than the activation energies of 0.95 eV and 0.66 eV for the competing desorption of CH_3_OH* and C_2_H_5_OH*, respectively. In contrast, after decorating the Mo-edge SVs with potassium, the activation energies for CH_3_OH* and C_2_H_5_OH* desorption are significantly decreased to 0.45 eV and 0.36 eV, respectively, which are markedly lower than the activation energies of 0.75 eV and 0.65 eV for C-O cleavage of CH_3_OH* and C_2_H_5_OH*, respectively. In addition, the difference between the free energies of desorption and dissociation states is also notably decreased after potassium decoration, making the desorption more favorable while dissociation less favorable in thermodynamics compared with those without potassium. Similar effects were also observed in the desorption of C_3_H_7_OH and C_4_H_9_OH (Fig. 4f). These results suggest that potassium modification has little effect on the activation energies for C-O cleavage as also evidenced by the similar apparent activation energies for the hydrogenation of CH_3_OH to CH_4_ and C_2_H_5_OH to C_2_H_6_ on the ER-MoS_2_ and ER-MoS_2_-K (Supplementary Fig. 25b, c), but has a significant effect in promoting the kinetics and thermodynamics for alcohols desorption, which contributes to the hindered deep-hydrogenation of alcohols (Supplementary Fig. 25) and thus the enhancement of alcohols selectivity.

The effect of potassium in promoting the formation and desorption of alcohols is also verified by the in-situ high pressure DRIFTS characterizations for the CO hydrogenation over the ER-MoS_2_ and ER-MoS_2_-K, in which the gradually enhanced vibration bands of alcohols can be observed on the ER-MoS_2_-K at elevated temperatures, whereas only the alkyl species was detected on the ER-MoS_2_ surface under identical reaction conditions (Supplementary Fig. 26). The facilitated desorption of alcohols on potassium modified Mo-edge SVs can be attributed to the electrostatic interaction between the potassium and the hydroxyl of alcohols, which weakens their adsorption on the Mo site, as reflected by the decreased charge density in the Mo-O bonding region when potassium exists (Fig. 4g). These results support that the potassium-decorated edge SVs of MoS_2_ are the active sites for the selective CO hydrogenation to higher alcohols.

## Discussion

The precise regulations of the activities for C-O cleavage and C-C coupling are always the challenging tasks in selective CO hydrogenation to specific C_2+_OH. In this study, we reveal the superiority of MoS_2_-edge SVs in facilitating simultaneously the C-O cleavage and C-C coupling, and the key role of potassium in promoting the timely desorption of alcohols via electrostatic interaction with hydroxyls of alcohols, thereby enabling the controlled carbon-chain growth for the preferential formation of C_2-4_OH. Highly selective CO hydrogenation to C_2-4_OH was achieved over potassium-modified edge-rich MoS_2_, which exhibits high CO conversion of 17% and C_2-4_OH selectivity of 45.2% in hydrogenated products at 240 °C and 50 bar, surpassing previously reported non-noble metal-based catalysts under similar conditions. In contrast, methanol was always the primary product at a wide range of reaction temperatures over the basal plane-dominated MoS_2_-based catalyst. This work presents the high flexibility of edge SVs of MoS_2_ in tailoring both C-O cleavage and C-C coupling for carbon-chain growth in CO hydrogenation, thus providing a prototype for the rational design of the nanostructure and microenvironment of active sites for selective hydrogenation reactions.

## Methods

### Preparation of catalysts

The Edge-rich MoS_2_ (ER-MoS_2_) was synthesized by using the SBA−15 as template. Typically, 400 mg (NH_4_)_6_Mo_7_O_24_·4H_2_O and 2.5 g SBA-15 were dispersed in 50 mL deionized water and followed by drying at room temperature under magnetic stirring. The obtained power was then dried overnight under 80 °C. The dried product and 10 mL CS_2_ were sealed into a 40 mL stainless steel autoclave under Ar protection and maintained at 400 °C for 4 h. After that, the HF solution treatment under room temperature for >8 h was conducted to remove SBA-15 template. The final product was then washed with water and absolute ethanol for several times and was dried at 80 °C.

The PD-MoS_2_ was synthesized by using the method of our previous work^40^. Typically, 900 mg (NH_4_)_6_Mo_7_O_24_·4H_2_O was dissolved in 20 mL deionized water to form a homogeneous solution. Then, the solution and 10 mL CS_2_ were sealed into a 40 mL stainless steel autoclave under Ar protection and maintained at 400 °C for 4 h. The product was treated with 6 mol L^−1^ KOH solution under stirring at 60 °C for 3 h, followed by washing with pure water and absolute ethanol for several times and then drying at 80 °C.

The ER-MoS_2_^foam^ and the PD-MoS_2_^foam^ were synthesized by using the SiO_2_ sphere as template. For the synthesis of SiO_2_ sphere, 300 mL ethanol, 100 mL deionized water and 28 mL NH_3_·H_2_O (aq.) were mixed. After 10 min of stir, 26 mL tetraethyl orthosilicate (TEOS) was added into the solution, followed by sealed drastic stirring for 5 h to form SiO_2_ sphere. The obtained SiO_2_ sphere was collected by centrifugation, and was washed by water and absolute ethanol for several times. After that, the product was dried overnight at 80 °C. For the synthesis of ER-MoS_2_^foam^, 400 mg (NH_4_)_6_Mo_7_O_24_·4H_2_O and 2.5 g SiO_2_ sphere were dispersed in 50 mL deionized water and followed by drying at room temperature under magnetic stirring. The obtained power followed the same reaction and treatment process as that of ER-MoS_2_. For the synthesis of PD-MoS_2_^foam^, 400 mg (NH_4_)_6_Mo_7_O_24_·4H_2_O, 800 mg thiourea and 2.5 g SiO_2_ sphere were dispersed in 50 mL deionized water and followed by drying at room temperature under magnetic stirring. The obtained power was then dried overnight under 80 °C. The dried product was sealed into a 40 mL stainless steel autoclave under Ar protection and maintained at 400 °C for 4 h. After that, the product followed the same treatment process as that of ER-MoS_2_.

MoS_2_ materials were decorated by potassium before catalytic test, which was used as a promoter without affecting the morphology and crystal form of MoS_2_. Typically, MoS_2_ was dispersed in 20 mL deionized water by ultraphonic for 30 min. Then, the K_2_CO_3_ (a.q.) was dispersed into the dispersion by ultraphonic for another 30 min, followed by drying at room temperature under magnetic stirring. The obtained product was then dried under 80 °C. The mole ratio of K/Mo was controlled in 0.2 unless otherwise stated.

### Evaluation of catalytic performance

The catalytic reactions were performed with a high-pressure fixed-bed reactor equipped with gas chromatograph (GC). Typically, before the reaction, 200 mg catalyst in grain sizes of 250-600 μm was pretreated in-situ in a H_2_ gas flow of 30 mL min^−1^ at 1 bar and a temperature of 300 °C for 1 ~ 3 h. After the reduction, the reactant was introduced into the reactor. Ar was mixed into the H_2_/CO mixture as an internal standard for the calculation of CO conversion. The reactions were operated under a pressure of 50 bar and temperatures range from 200 °C to 280 °C, with a H_2_/CO ratio of 2/1.

The methanol and ethanol hydrogenation experiments were also performed in high-pressure fixed-bed reactor with 100 mg catalyst. After the pre-reduction process as described above, liquid methanol or ethanol (0.005 mL min^−1^) was fed into the reactor using a liquid pump, meanwhile, high purity H_2_ was fed into the reactor at a gas flow rate of 30 mL min^−1^ at 1 bar and temperatures from 180 °C to 240 °C.

Products were analyzed by an online gas chromatograph, which was equipped with a thermal conductivity detector (TCD) and a flame ionization detector (FID). TDX-01 packed column was connected to TCD, and RT-Q-BOND-PLOT capillary column was connected to FID. The product selectivity was calculated on a molar carbon basis. The catalytic performances after 20 h of reaction were typically used for discussion.

The forward reaction rate (*r*_f_) was calculated on the basis of the measured net reaction rate (*r*_n_) and equilibrium factor (*η*):^41^

1 $${r}_{{{{{{\rm{f}}}}}}}={r}_{{{{{{\rm{n}}}}}}}/(1-\eta )$$

As an example, for the reaction CO (g) + 2H_2_ (g) ⇌ CH_3_OH (g), *η* was calculated using

2 $$\eta=\frac{{P}_{{{{\mbox{CH}}}}_{3}{{\mbox{OH}}}}}{{P}_{{{\mbox{CO}}}}\times {{{\mbox{P}}}}_{{{{\mbox{H}}}}_{2}}^{2}}\times \frac{1}{K}$$

Where *P*_*x*_ is the partial pressure of species *x* (in atm) and *K* is the equilibrium constant for the reaction, which is 3.271 × 10^−3^ atm^–2^ at 240 °C. The calculated forward reaction rates in this work are almost identical to the net reaction rates.

### Catalyst characterization

Transmission electron microscopy (TEM), High-angle annular dark field scanning transmission electron microscopy (HAADF-STEM) and energy-dispersive X-ray (EDX) mapping measurements were carried out on a Phillips Analytical FEI Tecnai20 electron microscope or on a JEOL ARM-200F field-emission transmission electron microscope operated at an acceleration voltage of 200 kV. Scanning electron microscopy (SEM) measurements were carried out using a Hitachi S-4800 scanning electron microscope with a 15 kV accelerating voltage.

X-ray fluorescence (XRF) characterizations were conducted on a Zetium XRF spectrometer. Prior to measurement, the sample was pretreated with H_2_ at 300 °C for 1 h and subsequently purged with Ar at 25 °C overnight. Inductively coupled plasma optical emission spectrometry (ICP-OES) was conducted on Shimadzu ICPS-8100. The C and S contents in the used catalysts were analyzed using a EMIA-8100 elemental analyzer.

X-ray diffraction (XRD) patterns were recorded on a Rigaku Ultima IV diffractometer with Cu Kα radiation (λ = 0.15406 nm) at 40 kV and 30 mA. The diffraction angles were scanned from 5 to 85 degrees (2θ) with a speed of 10-degree min^−1^.

X-ray absorption fine structure (XAFS) spectra were measured at the BL14W1 beamline of the Shanghai Synchrotron Radiation Facility (SSRF).

N_2_ adsorption measurements were performed on Quadrasorb evo. Prior to N_2_ adsorption, the samples were degassed under vacuum at 120 °C for 6 h.

NH_3_ temperature-programmed desorption (NH_3_-TPD) and NO chemisorption measurements were performed on a Micromeritics Auto Chem II 2920 instrument. Prior to each measurement, the sample was pretreated in situ with H_2_ at 300 °C for 3 h and subsequently purged with He at 300 °C for 3 h. For NH_3_-TPD, the adsorption of NH_3_ was performed at 50  °C in He gas containing 10% NH_3_ for 1 h and TPD was performed in He flow by raising the temperature to 800 °C with a rate of 10 °C min^−1^. The NO chemisorption experiments were performed at 40 °C by pulsing 0.5 mL of 2% NO/He (0.314 µmol NO per pulse) through the catalyst every 30 min^30,42^. The NO effluent (detected by mass spectrometer) gradually increased to a constant value, signifying NO saturation of the catalyst, and then the total uptake of NO was calculated.

X-ray photoelectron spectroscopy (XPS) characterizations were performed on ThermoFisher ESCALAB 250Xi spectrometer or SPECS spectrometer using Al Kα x-rays as the excitation source. XPS characterizations of the used catalysts after ~1000 h of on-stream reaction were conducted without exposing the samples to air.

Characterizations using the hyphenated technology of in-situ time-resolved energy dispersive-X-ray absorption spectroscopy (ED-XAS) and in-situ diffuse reflectance infrared Fourier transform spectroscopy (DRIFTS) were performed at D-line (BL05U&BL06B1) of Shanghai Synchrotron Radiation Facility. Before measurement, MoS_2_ catalyst powder was loaded into the cell of in-situ reactor. Then, a mixture gas of 36%H_2_/Ar was introduced into the reactor. After that, the reactor was heated to 300 °C with a rate of 5 °C min^−1^, and was held at 300 °C for 1 h. Spectra were recorded during the whole in-situ experiment.

Quasi in-situ electron paramagnetic resonance (EPR) spectroscopic measurements were performed on a Bruker A200 EPR spectrometer operated at X-band frequency with a microwave frequency of 9.32 GHz, a microwave power of 10 mW and a modulation frequency of 100 kHz. EPR characterization was conducted using a quasi in-situ method, in which the sample was reduced in the sample tube, and then the sample tube was transferred in EPR spectrometer for measurement. Sample was not exposed to air during the whole process. Typically, catalyst powder was placed in a quartz EPR sample tube. Before measurement, the catalyst was pretreated by H_2_ for 3 h at 300 °C, and then was purged with Ar at 300 °C for 1 h. All spectra were normalized with the mass of catalysts.

In-situ diffuse reflectance infrared Fourier transform spectroscopy (DRIFTS) measurements were performed using a Fourier transform infrared spectrometer (Nicolet 6700 for atmospheric pressure test and vertex 70 V for high pressure test) equipped with a mercury cadmium telluride detector. The in-situ DRIFT spectra were recorded by collecting 64 scans at a resolution of 4 cm^−1^. Before measurement, all catalysts were pretreated in-situ in a H_2_ gas flow of 30 mL min^−1^ at 1 bar and a temperature of 300 °C for 1 h. For in-situ DRIFTS measurement of CO hydrogenation at atmospheric pressure, catalysts were subsequently cooled down to 25 °C in H_2_ flow after H_2_ pretreatment, then the background spectra were obtained. After that, catalysts were exposed to syngas (CO/H_2_ = 1/2) in a gas flow of 30 mL min^−1^ with a pressure of 1 bar. For in-situ DRIFTS measurement of methanol adsorption at atmospheric pressure, after H_2_ pretreatment, catalysts were purged with Ar for 30 min and subsequently cooled down to 25 °C in Ar flow, then the background spectra were obtained. After that, catalysts were exposed to methanol steam, which was introduced by Ar. For in-situ DRIFTS measurement of CO hydrogenation at high pressure, catalysts were subsequently cooled down to reaction temperature in H_2_ flow after H_2_ pretreatment, then the background spectra were obtained. After that, catalysts were exposed to syngas (CO/H_2_ = 1/2) in a gas flow of 30 mL min^−1^ with a pressure of 30 bar. Spectra were recorded during the whole in-situ DRIFTS experiment.

In-situ Synchrotron-based VUV photoionization mass spectrometry (SVUV-PIMS) study was carried out at the mass spectrometry end-station of the National Synchrotron Radiation Laboratory at Hefei, China^43,44^. Before experiment, 200 mg catalysts were treated in-situ by H_2_ at 300 °C for 1 h. To detect the intermediates and products during CO hydrogenation, the reduced catalysts were exposed to CO/H_2_ (1/2) atmosphere with a flow rate of 50 mL min^−1^ and a pressure of 5 bar at 240 °C, the photoionization mass spectra were collected for 300 s at a photon energy of 11 eV.

Quasi in-situ synchrotron radiation photoelectron spectroscopy (SRPES) measurements were performed at the photoemission end-station at the beamline BL10B of the National Synchrotron Radiation Laboratory (NSRL) in Hefei, China. The beamline is connected to a bending magnet and covers photon energies from 100 to 1000 eV with a resolving power (*E*/Δ*E*) better than 1000. The end-station is composed of four chambers: an analysis chamber, a preparation chamber, a load-lock chamber and a high-pressure reactor. The analysis chamber, with a base pressure of <2 × 10^−10^ torr, is connected to the beamline with a VG Scienta R3000 electron energy analyzer and a twin-anode X-ray source. Before experiment, the catalysts were treated in-situ by H_2_ at 300 °C for 1 h. Then, the catalysts were treated with reaction gas (H_2_/CO = 2) at 240 °C and 5 bar for 0.5 h, followed by transferring to the analysis chamber under high vacuum for SRPES measurement.

### Computational details

All DFT calculations were performed using the Vienna Ab initio Simulation Package (VASP)^45–48^ and the Atomic Simulation Environment (ASE)^49^. The projector augment wave (PAW) method with Perdew-Burke-Ernzerhof (PBE) functional for exchange-correlation term was used with a cutoff energy of 400 eV^50–53^. The Brillouin zone was sampled with a Monkhorst-Pack 1 × 1 × 1 *k*-point for all models^54^. Zero damping DFT-D3 method of Grimme was used to correct van der Waals interactions^55,56^. The models of a 6 × 6 × 1 tri-layer MoS_2_ supercell and a nanoribbon MoS_2_ with eight repeating units along ribbon direction, saturated with S monomers were built for simulating in-plane and Mo-edge configurations, respectively, with vacuum region about 15 Å between slabs or ribbons. The energy convergence was set to 1 × 10^−5^ eV in structural optimizations. Fix-bond-length method in ASE was applied to search transition states and a tolerance of 0.06 eV/Å was set for force convergence. The free energies of gaseous molecules were calculated as: ${E}_{{{\mbox{total}}}}+{{\mbox{ZPE}}}+H-{TS}+{RT}{{{{\mathrm{ln}}}}}\frac{P}{{P}_{0}}$, where ${E}_{{{\mbox{total}}}}$ is DFT calculated total energy, ZPE is the zero-point energy, *H* and *S* are enthalpy and entropy based on ideal gas approximation, *T* is the reaction temperature, *R* is ideal gas constant (8.314 J mol^−1^ K^−1^) and *P* and *P*_0_ are partial pressure of specific gas components and standard atmosphere pressure, respectively. ${E}_{{{\mbox{total}}}}+{{\mbox{ZPE}}}+{H}_{{{\mbox{vib}}}}-T{S}_{{{\mbox{vib}}}}$, where *H*_vib_ and *S*_vib_ are enthalpy and entropy parts of non-imaginary vibrations based on harmonic approximation, was implemented to calculate the free energies of reaction intermediates and transition states.

### Reporting summary

Further information on research design is available in the Nature Portfolio Reporting Summary linked to this article.

### Supplementary information

Supplementary Information

Peer Review File

Reporting Summary

### Source data

Source Data

## Data Availability

All data supporting this work are available in the main text and Supplementary Information. Source data are provided with this paper.
