# Supplementary material for: Edge-rich molybdenum disulfide tailors carbon-chain growth for selective hydrogenation of carbon monoxide to higher alcohols
Source: Nat Commun. 2023 Oct 26;14:6808. doi: 10.1038/s41467-023-42325-z (PMC10603039; doi:10.1038/s41467-023-42325-z)
Supplement: Supplementary file 1 — Supplementary Information [file 41467_2023_42325_MOESM1_ESM.pdf]

Edge-rich molybdenum disulfide tailors carbon-chain growth for selective hydrogenation of carbon monoxide to higher alcohols

Authors: Jingting Hu[1,2,7], Zeyu Wei[1,3,7], Yunlong Zhang[1,3], Rui Huang[1], Mingchao Zhang[2], Kang Cheng[2], Qinghong Zhang[2], Yutai Qi[1,2], Yanan Li[1,2], Jun Mao[1,2], Junfa Zhu[4], Lihui Wu[4], Wu Wen[4], Shengsheng Yu[4], Yang Pan[4], Jiuzhong Yang[4], Xiangjun Wei[5], Luozhen Jiang[6], Rui Si[6], Liang Yu[1,3]*, Ye Wang[2]* & Dehui Deng[1,2,3]*

[1]State Key Laboratory of Catalysis, Collaborative Innovation Center of Chemistry for Energy Materials, Dalian Institute of Chemical Physics, Chinese Academy of Sciences, Dalian 116023, China.

[2]State Key Laboratory of Physical Chemistry of Solid Surfaces, Collaborative Innovation Center of Chemistry for Energy Materials, College of Chemistry and Chemical Engineering, Xiamen University, Xiamen 361005, China.

[3]University of Chinese Academy of Sciences, Beijing 100049, China.

[4]National Synchrotron Radiation Laboratory, University of Science and Technology of China, Hefei 230029, China.

[5]Shanghai Synchrotron Radiation Facility, Shanghai Advanced Research Institute, Chinese Academy of Sciences, Shanghai 201204, China.

[6]Shanghai Institute of Applied Physics, Chinese Academy of Sciences, Shanghai 201204, China.

[7]These authors contributed equally to this work.

*Correspondence to: lyu@dicp.ac.cn; wangye@xmu.edu.cn; dhdeng@dicp.ac.cn

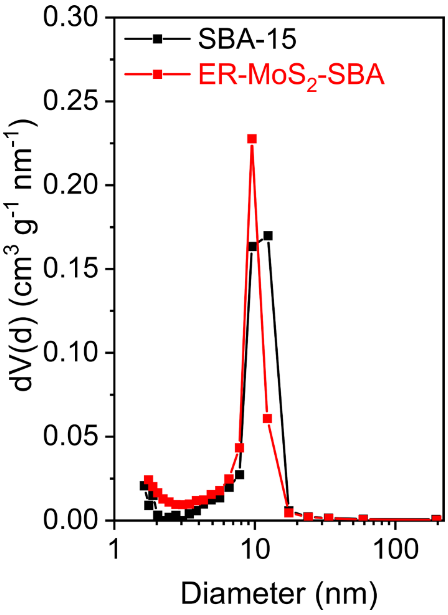

Supplementary Fig. 1. Pore-size distribution curves of SBA-15 template and ER-MoS_2-SBA. These curves were derived from the adsorption branches of the isotherms by using the Barrett-Joyner-Halenda (BJH) method.

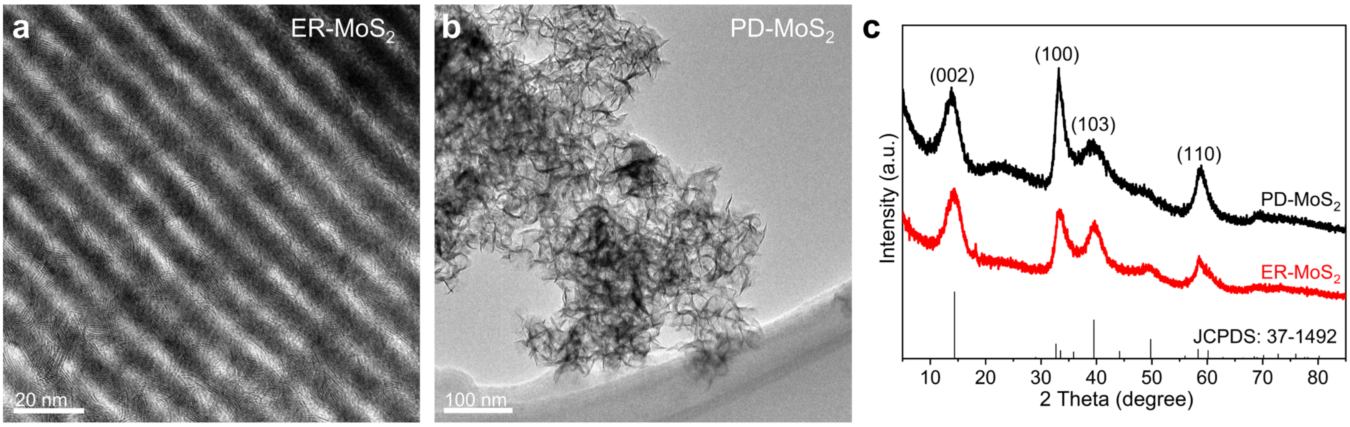

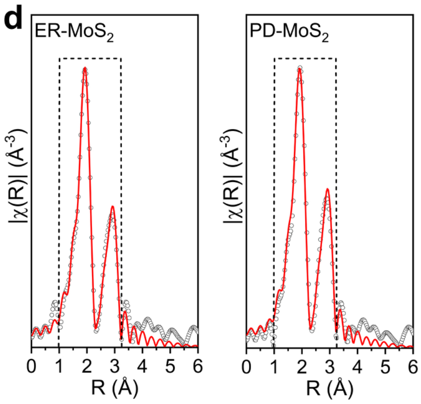

e

Sample	shell	*CN**	*R* (Å)	σ^2 (10^{-3} Å^2)	ΔE_0 (eV)	R factor
PD-MoS_2	Mo-S	5.58±0.22	2.41±0.00	0.0041±0.0002	2.7±0.2	0.0102
	Mo-Mo	5.63±0.44	3.17±0.00	0.0053±0.0003		
ER-MoS_2	Mo-S	6.19±0.24	2.41±0.00	0.0034±0.0002	2.6±0.3	0.0076
	Mo-Mo	5.02±0.46	3.17±0.00	0.0043±0.0003		

Supplementary Fig. 2. a, b, TEM images of ER-MoS_2 and PD-MoS_2. **c,** XRD patterns of ER-MoS_2 and PD-MoS_2. **d,** Mo K-edge EXAFS fitting results for ER-MoS_2 and PD-MoS_2 in *R* space. The circles show the original data, and the red curve is the fitting curve, the areas enclosed by dotted black lines represent the fitting region. **e,** Mo K-edge EXAFS fitting results of the PD-MoS_2 and the ER-MoS_2. **CN*, coordination number.

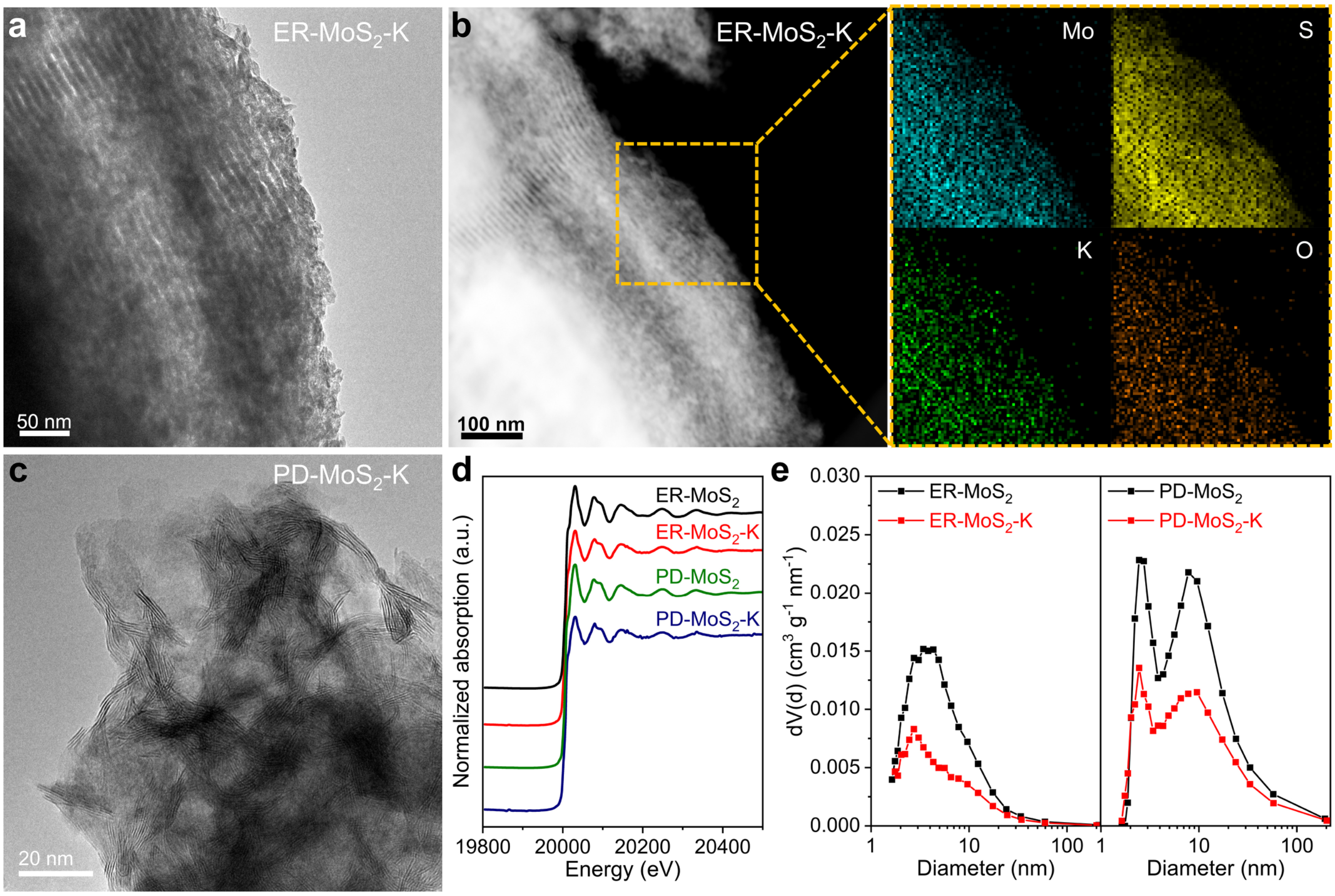

Supplementary Fig. 3. a, TEM image of the used ER-MoS_2-K catalyst. **b,** HAADF-STEM image and EDX mapping of the used ER-MoS_2-K catalyst. **c,** TEM image of the used PD-MoS_2-K catalyst. **d,** XAS spectra of the fresh ER-MoS_2, PD-MoS_2, and the used ER-MoS_2-K, PD-MoS_2-K catalysts. **e,** Pore-size distribution curves of ER-MoS_2, ER-MoS_2-K, PD-MoS_2, and PD-MoS_2-K. These curves were derived from the adsorption branches of the isotherms by using the Barrett-Joyner-Halenda (BJH) method.

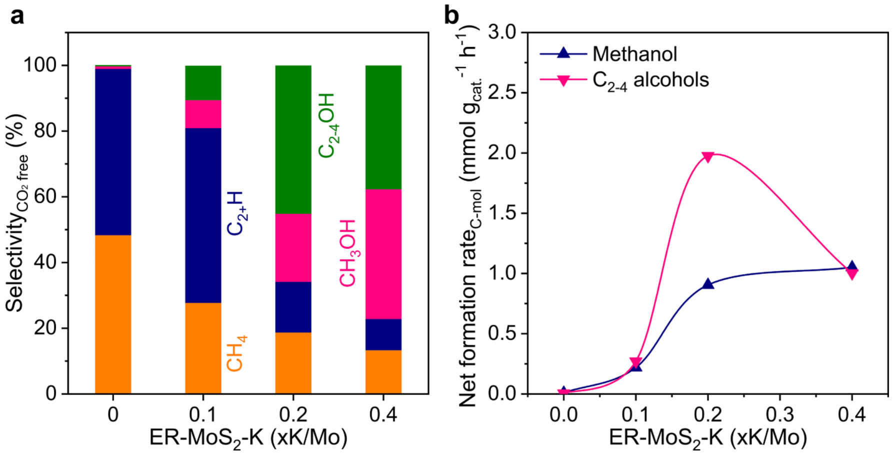

Supplementary Fig. 4. a, Distribution of CO hydrogenation products over the ER-MoS_2-K catalysts with different potassium content (x is the mole ratio of K/Mo). The product selectivity was calculated on a CO_2-free basis. **b,** Evolutions of net formation rate of methanol and C_{2-4} alcohols with increasing K/Mo ratio. Catalysts were pretreated in-situ by H_2 at 300 °C for 1 hour before reaction. Reaction activity tests were performed at 50 bar, 240 °C, 3000 mL $g_{cat.}^{-1}$ h^{-1} and H_2/CO ratio of 2.

Note: The alcohols selectivity of ER-MoS_2-K catalysts increases with increasing potassium content, and the highest selectivity and formation rate of $C_{2-4}OH$ are achieved at a K/Mo mole ratio of 0.2.

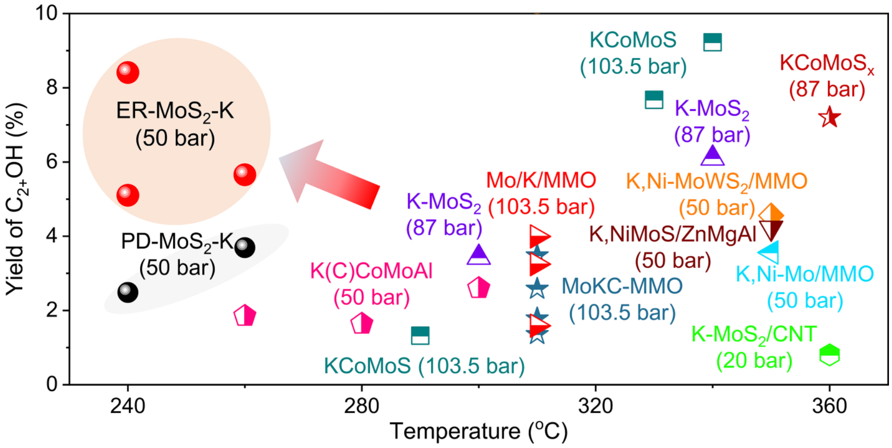

Supplementary Fig. 5. Comparison in $C_{2+}OH$ yield over ER-MoS_2-K, PD-MoS_2-K and other MoS_2-based catalysts reported in literatures (see Supplementary Table 4 for more details). Catalysts were pretreated in-situ by H_2 at 300 °C for 1 hour before reaction. Reaction activity tests were performed using a tubular fixed-bed reactor at 50 bar and a H_2/CO of 2.

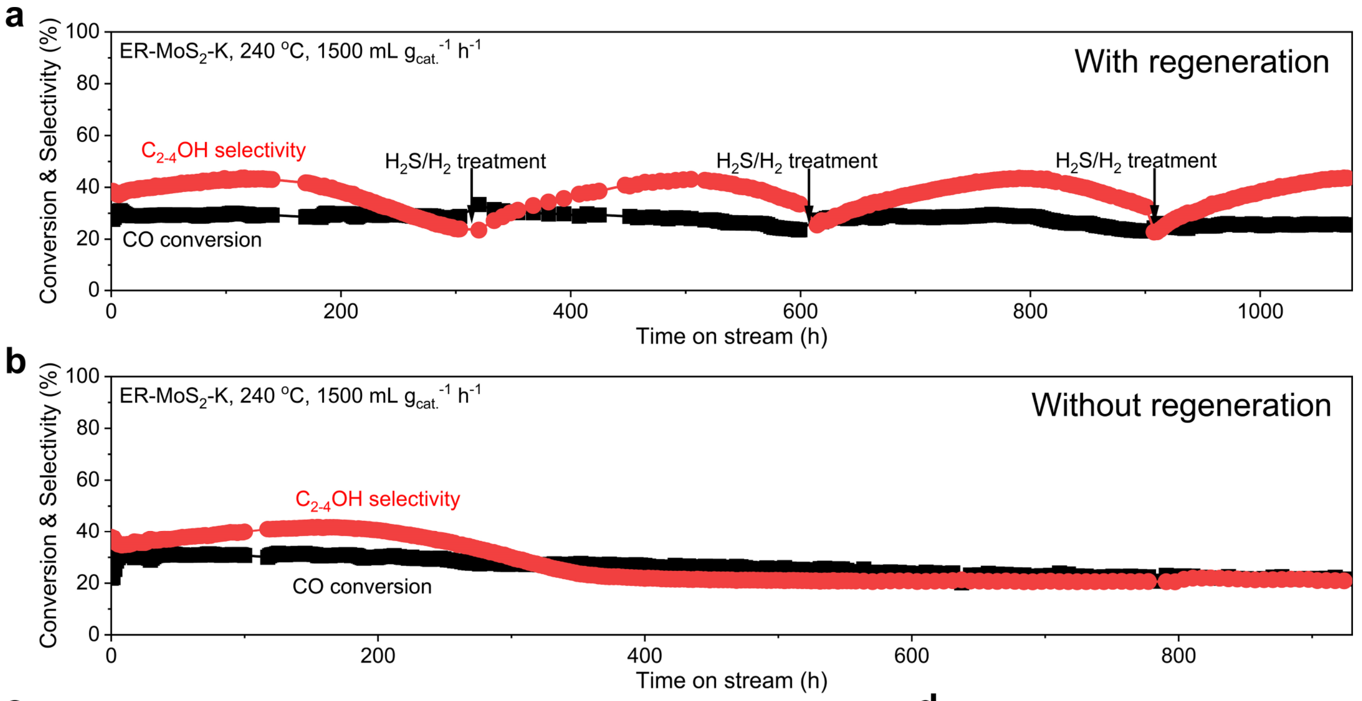

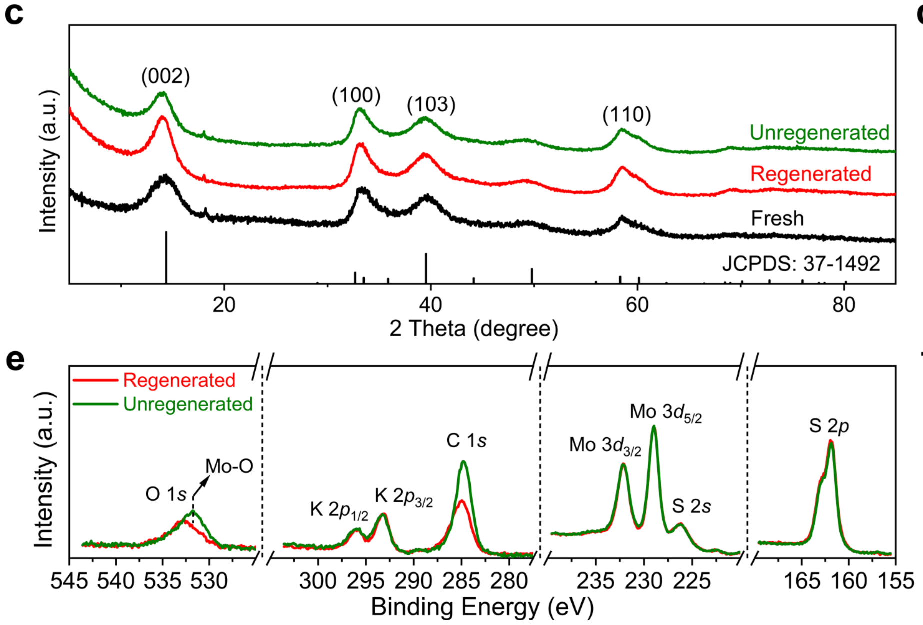

d

Sample	C%	S%
Fresh	5.4	45.2
Regenerated	6.1	36.8
Unregenerated	8.1	31.6

f

Sample	Surface S/Mo ratio
Regenerated	2.1
Unregenerated	1.9

Supplementary Fig. 6. a, b, Long-term performance test of CO hydrogenation over the ER-MoS_2-K catalyst. The catalyst in (**a**) was regularly treated with 5%H_2S/H_2 mixture at 300 °C for 3 hours at every TOS of 300 hours. **c,** XRD patterns of the fresh ER-MoS_2-K catalyst and the used catalyst after ~1000 hours of on-stream reaction. **d,** The C and S contents in the used catalysts quantified by using elemental analyzer. **e,** XPS spectra of the used catalysts after ~1000 hours of on-stream reaction. **f,** S/Mo atomic ratios in the used ER-MoS_2-K catalysts, which were calculated based on the sensitivity factor and the peak area of the Mo 3*d* and S 2*p*.

Note: In the absence of S in the feed gas, the $C_{2-4}OH$ selectivity decreases as the time-on-stream (TOS) is over 300 hours and then becomes stable to be around 21% (Supplementary Fig. 6b). However, by regularly treating

the catalyst with 5%H_2S/H_2 mixture at 300 ºC for 3 hours for every TOS of 300 hours, the catalytic performance can be recovered and then be well-maintained in a long-term stability test for 1075 hours (Supplementary Fig. 6a), indicating the high durability of the ER-MoS_2-K catalyst. XRD characterizations show that both the regenerated and unregenerated ER-MoS_2-K catalysts maintain the hexagonal 2H-MoS_2 crystal phase after the long-term reaction (Supplementary Fig. 6c). Quantifications of the C and S elements in the used catalysts by using elemental analyzer show that the unregenerated catalyst possesses a notably lower S content but higher C content than that in the regenerated one (Supplementary Fig. 6d), which agrees well with the XPS characterizations, showing the lower S/Mo ratio and higher intensity of surface C species and Mo-O species on the unregenerated catalyst (Supplementary Figs. 6e, f). Thus, we speculate that the deactivation of the ER-MoS_2-K catalyst could be due to the loss of S and the deposition of C and O during the reaction.

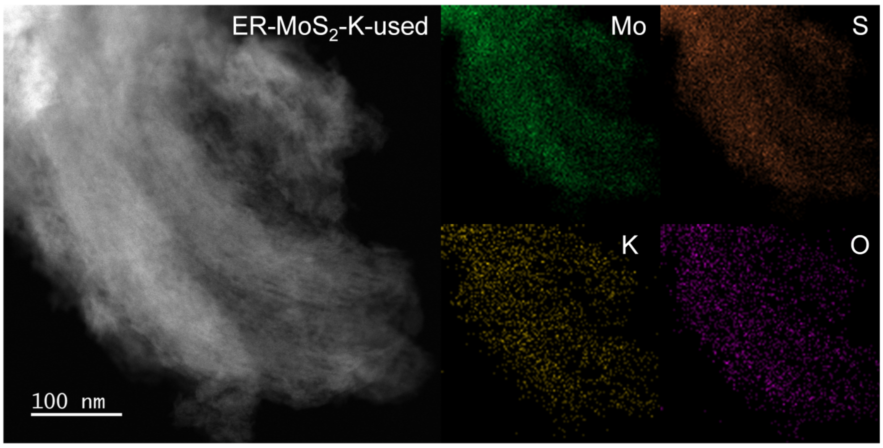

Supplementary Fig. 7. HAADF-STEM image and EDX mapping of the used ER-MoS_2-K catalyst with regeneration treatment after 1075 hours of on-stream reaction.

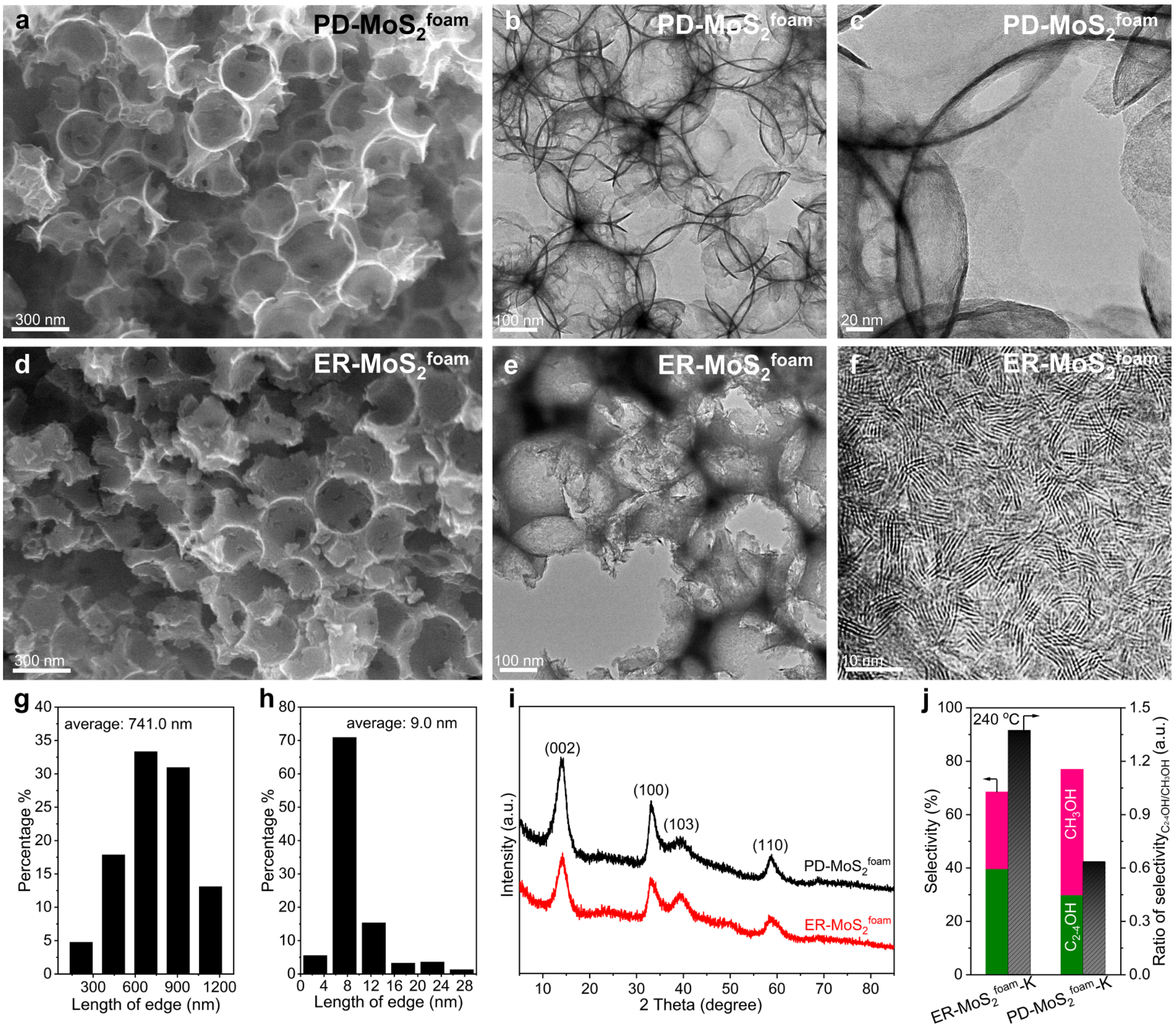

Supplementary Fig. 8. a, SEM image of PD-MoS_2^{foam}-K. **b, c,** TEM images of PD-MoS_2^{foam}-K. **d,** SEM image of ER-MoS_2^{foam}-K. **e, f,** TEM images of ER-MoS_2^{foam}-K. **g, h,** Edge-length statistics of PD-MoS_2^{foam}-K (**g**) and ER-MoS_2^{foam}-K (**h**) based on TEM results, respectively. **i,** XRD patterns of PD-MoS_2^{foam}-K and ER-MoS_2^{foam}-K. **j,** Alcohol distribution over PD-MoS_2^{foam}-K and ER-MoS_2^{foam}-K catalysts at 240 °C, 3000 mL $g_{cat.}^{-1}$ h^{-1}, 50 bar and H_2/CO of 2. The product selectivity was calculated on a CO_2-free basis. Catalysts were pretreated in-situ by H_2 at 300 °C for 1 hour before reaction.

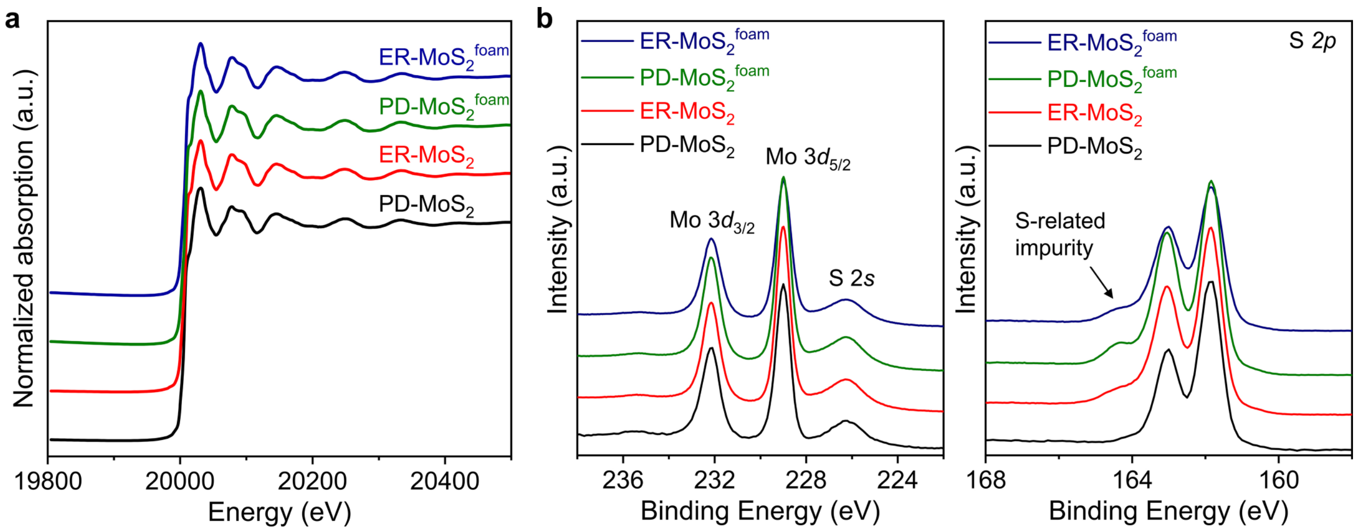

Supplementary Fig. 9. Mo K-edge XAS spectra (**a**) and XPS spectra (**b**) of different MoS_2 samples. The sulfur-related impurity can be fully removed during CO hydrogenation reaction and thus is not an active species (Supplementary Fig. 6e).

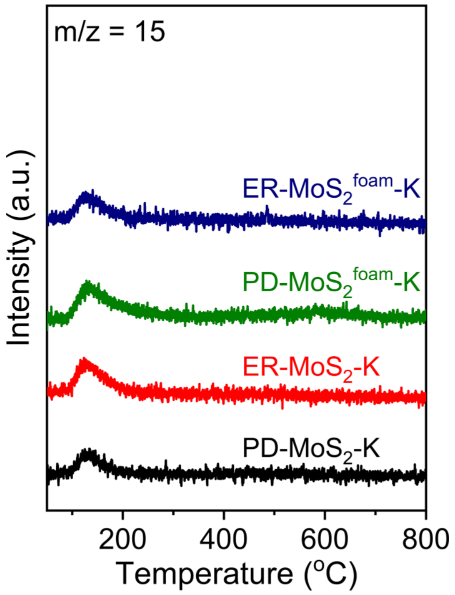

Supplementary Fig. 10. NH_3-TPD of PD-MoS_2-K, ER-MoS_2-K, PD-MoS_2^{foam}-K, and ER-MoS_2^{foam}-K catalysts. Prior to each measurement, the sample was pretreated in situ with H_2 at 300 °C for 3 h and subsequently purged with He at 300 °C for 3 h. The adsorption of NH_3 was performed at 50 °C in He gas containing 10% NH_3 for 1 h and TPD was performed in He flow by raising the temperature to 800 °C with a rate of 10 °C min^{-1}.

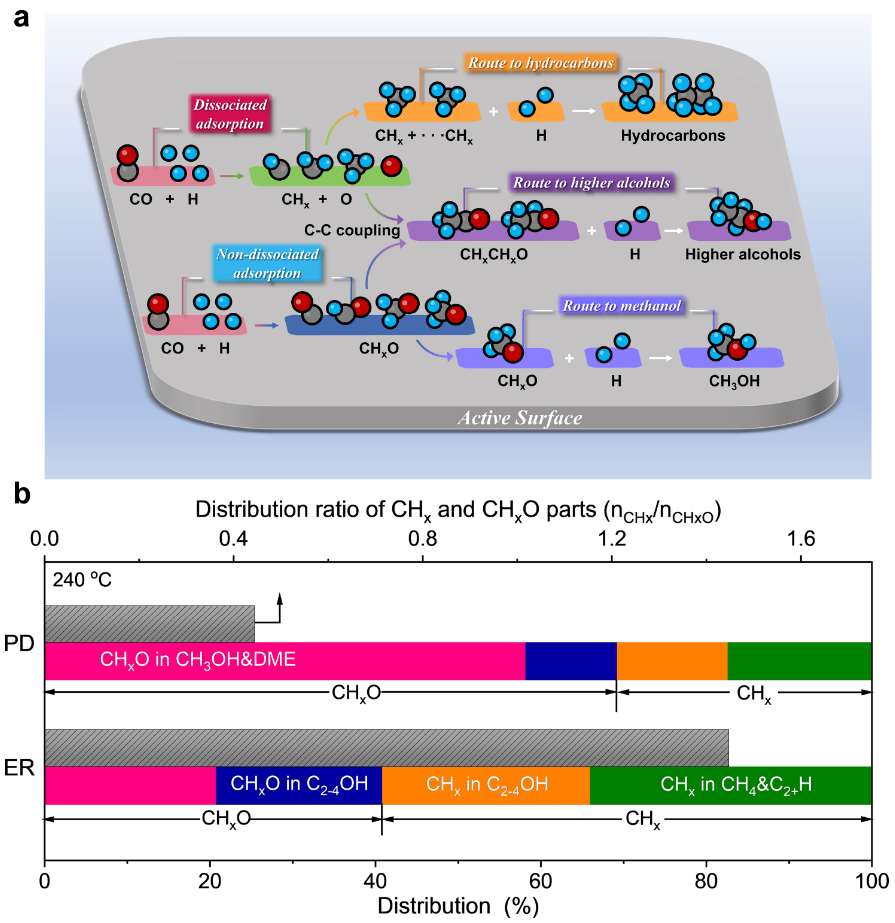

Supplementary Fig. 11. a, Typical reaction mechanism for the synthesis of higher alcohols, hydrocarbons, and methanol from CO hydrogenation. **b,** Comparison in the percentages of CH_xO ingredient and CH_x ingredient during reaction process over the ER-MoS_2-K and the PD-MoS_2-K. Selectivity was calculated on a CO_2-free basis.

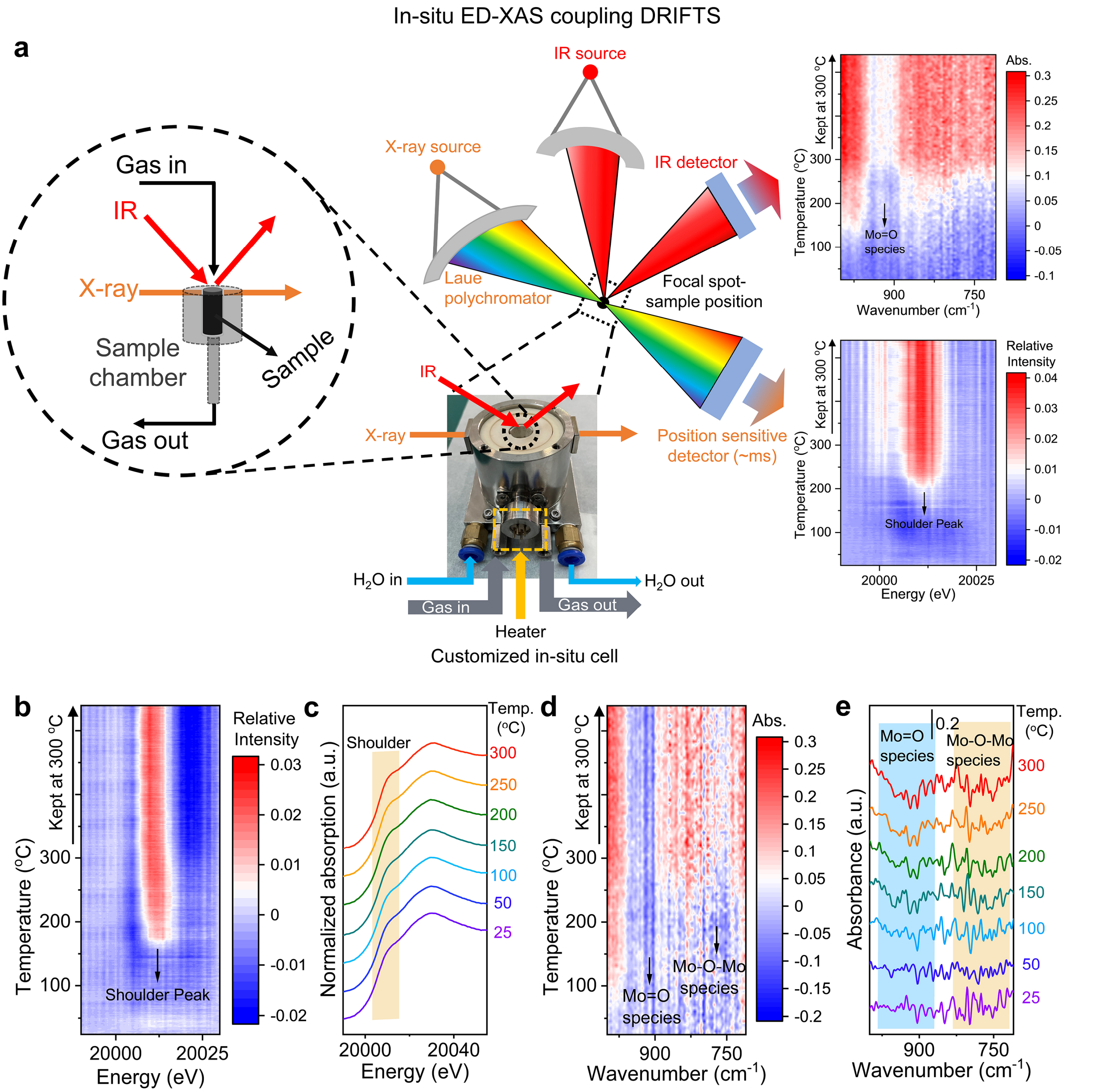

Supplementary Fig. 12. a, Schematic diagram of the hyphenated technology of the in-situ time resolved ED-XAS coupling in-situ DRIFTS characterizations. The inserted spectra are the same as those in Fig. 3a, c. **b-e,** In-situ ED-XAS (**b, c**) coupling in-situ DRIFTS (**d, e**) characterizations of PD-MoS_2-K during H_2 pretreatment measured with a hyphenated technology. Relative intensity in (**b**) was obtained by subtracting each spectrum to the first spectrum.

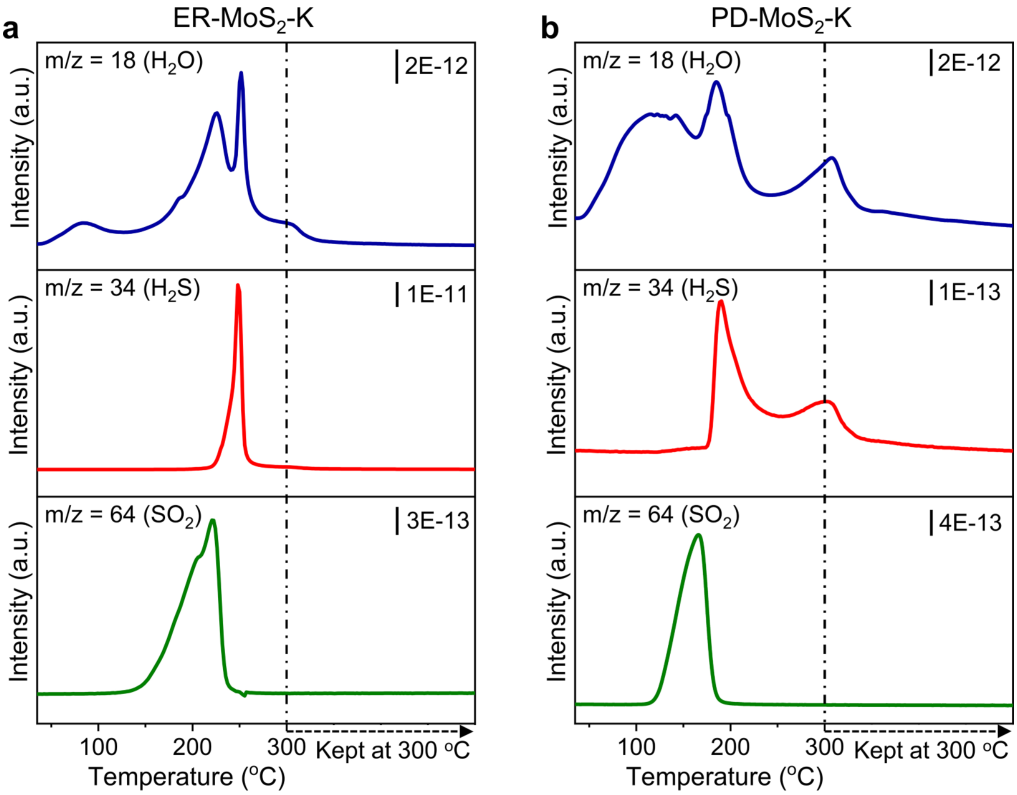

Supplementary Fig. 13. In-situ MS detection of the H_2 pretreatment products on ER-MoS_2-K (**a**) and PD-MoS_2-K (**b**).

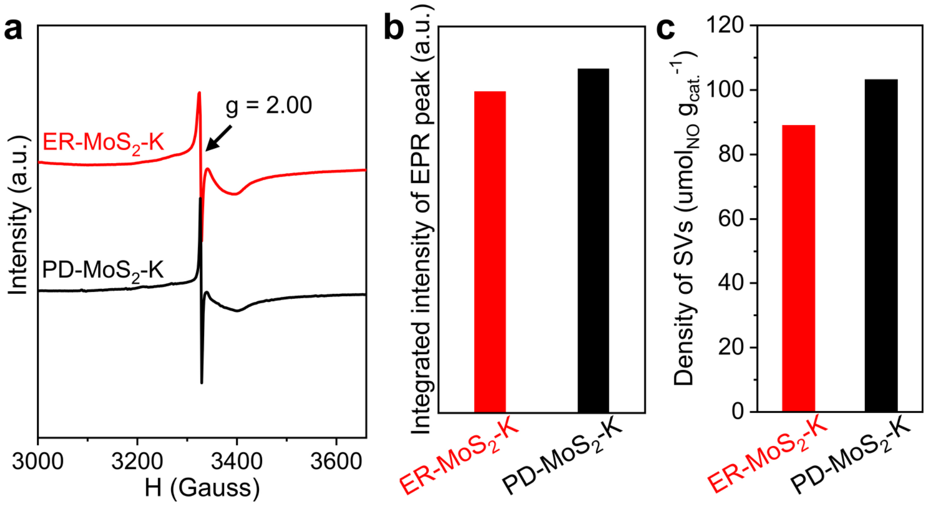

Supplementary Fig. 14. a, b, EPR spectra (**a**) and integrated intensity of EPR peak (**b**) of ER-MoS_2-K and PD-MoS_2-K. **c,** Densities of SVs on ER-MoS_2-K and PD-MoS_2-K quantified by using in-situ NO pulse adsorption.

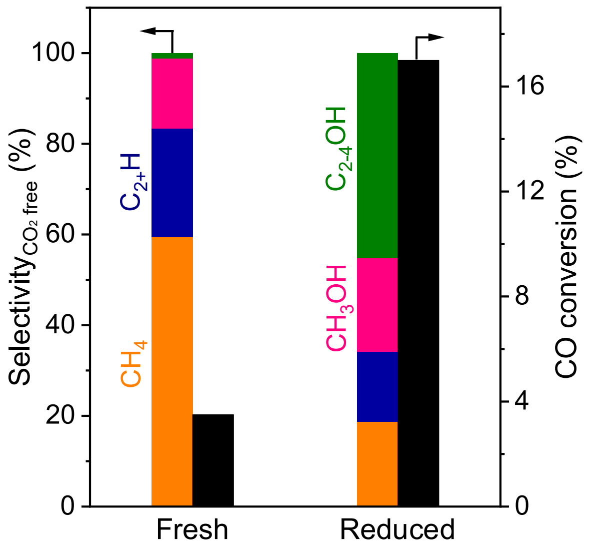

Supplementary Fig. 15. Catalytic performances of the ER-MoS_2-K catalyst before (fresh) and after (reduced) H_2 pretreatment at 300 °C for 1 h. Reaction activity tests were performed at 50 bar, 240 °C, 3000 mL $g_{cat.}^{-1}$ h^{-1}, and H_2/CO ratio of 2.

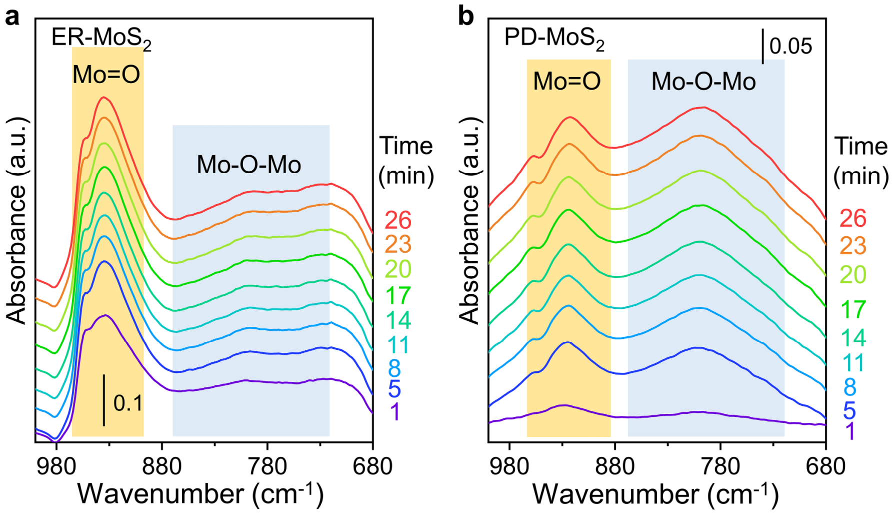

Supplementary Fig. 16. In-situ DRIFTS spectra of the ER-MoS_2 (**a**) and PD-MoS_2 (**b**) during the O_2 adsorption process.

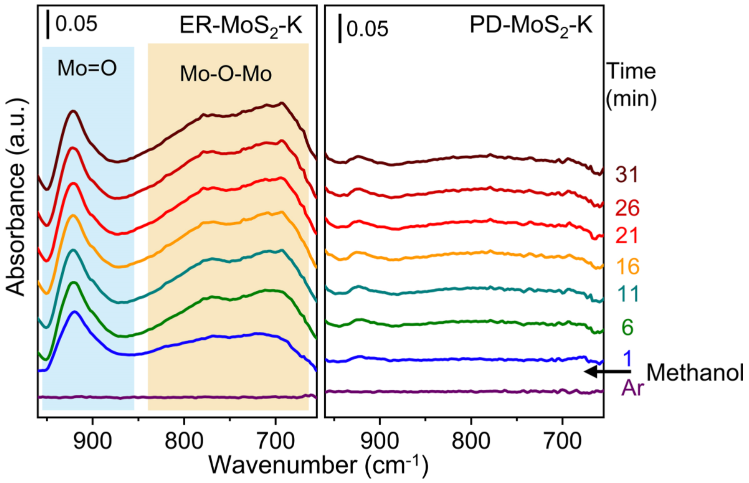

Supplementary Fig. 17. In-situ DRIFT spectra of methanol adsorption over the H_2-pretreated catalysts at 25 ºC.

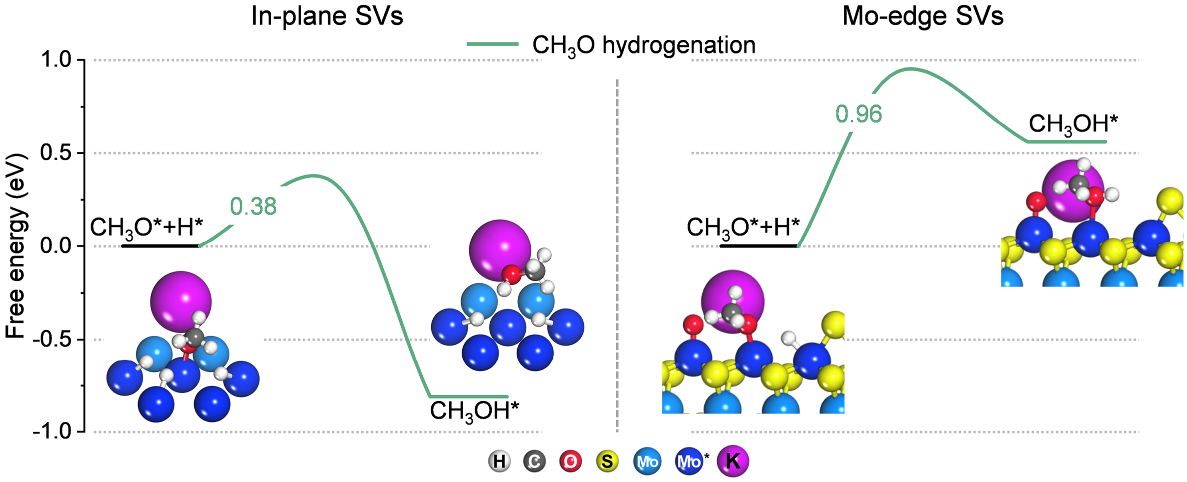

Supplementary Fig. 18. DFT calculated reaction barrier for the hydrogenation of CH_3O^* to CH_3OH on potassium-modified in-plane and edge double-Sv, respectively.

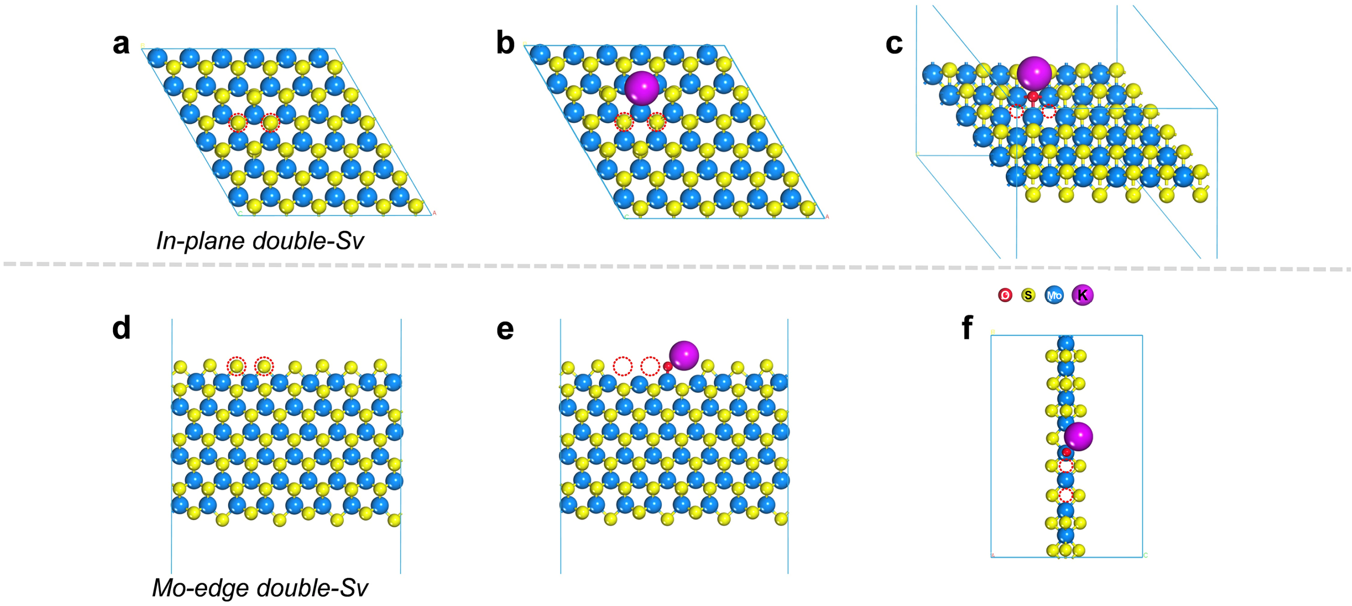

Supplementary Fig. 19. a-c, A tri-layer model of MoS_2 for simulating the in-plane sulfur vacancies. **d-f,** A nanoribbon model of MoS_2 for simulating the edge S vacancies. The dotted red circles denote the S atoms to be removed for creating the vacancies.

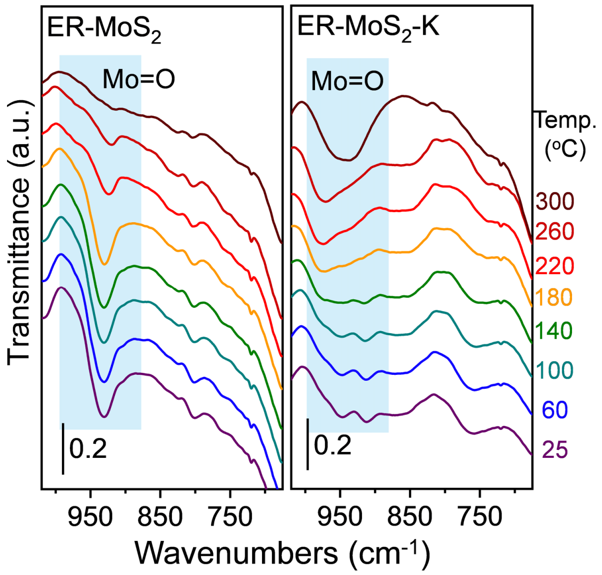

Supplementary Fig. 20. In-situ DRIFT spectra of ER-MoS_2 and ER-MoS_2-K during H_2 reduction.

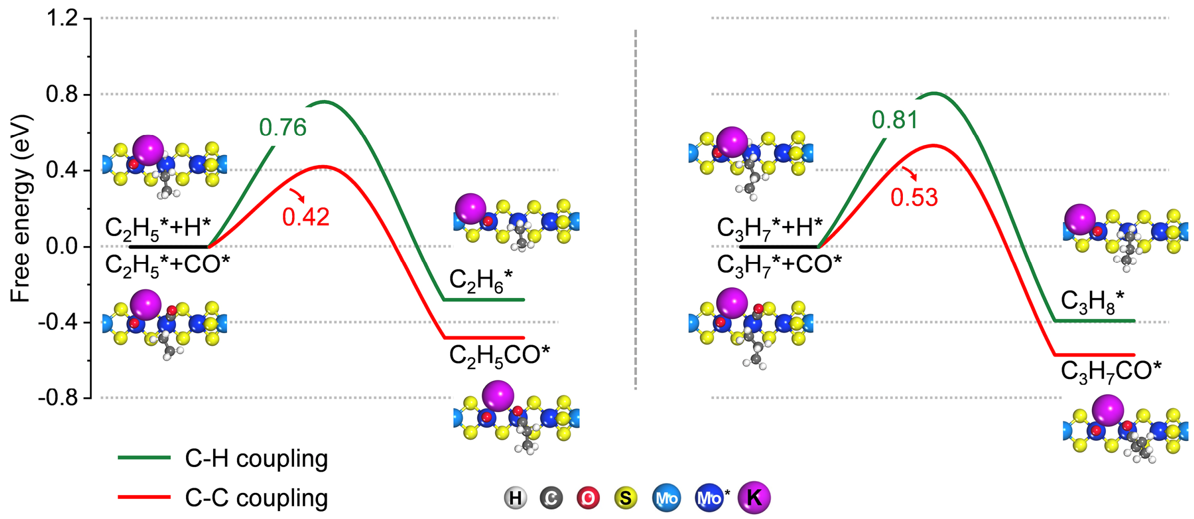

Supplementary Fig. 21. CO^* insertion barrier and hydrogenation barrier of alkyl species on potassium-modified edge double-Sv of MoS_2.

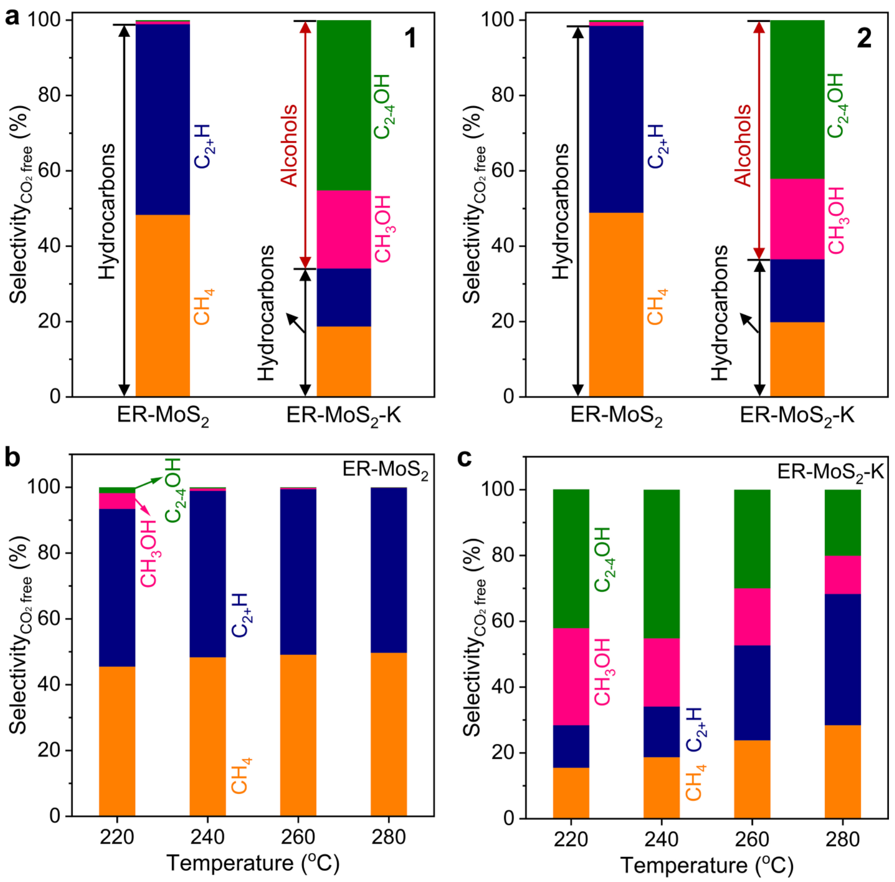

Supplementary Fig. 22. a, Distribution of CO hydrogenation products over two batches of individually synthesized ER-MoS_2 and ER-MoS_2-K at 240 °C, 3000 mL $g_{cat.}^{-1}$ h^{-1}, 50 bar and H_2/CO of 2. **b, c,** Distribution of CO hydrogenation products over ER-MoS_2 (**b**) and ER-MoS_2-K (**c**) at different reaction temperatures. The product selectivity was calculated on a CO_2-free basis. Catalysts were pretreated in-situ by H_2 at 300 °C for 1 hour before reaction. Reaction activity tests were performed at 50 bar, 3000 mL $g_{cat.}^{-1}$ h^{-1}, and H_2/CO ratio of 2.

Note: At reaction temperatures ranging from 220 to 280 °C, hydrocarbons are always the primary product with a selectivity of > 90% over the ER-MoS_2 catalyst, while the selectivity toward alcohols is significantly enhanced by decorating the ER-MoS_2 with K promoter. Such a phenomenon was also observed in previous studies[1,2], in which several possible reasons including the change of the electronic properties of MoS_2, the stabilization of alkoxy species, and the increase of the basicity of the catalyst, were speculated to explain the increase of alcohol selectivity with the decoration of K but without consensus on this effect.

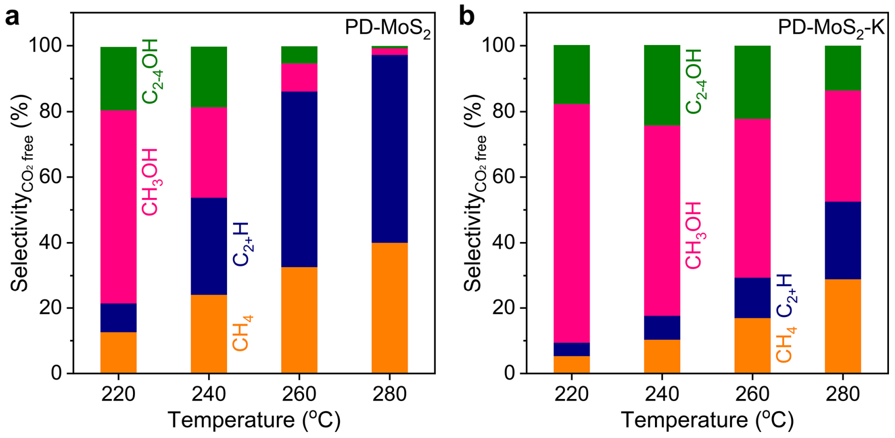

Supplementary Fig. 23. a, b, Distribution of CO hydrogenation products over PD-MoS_2 (**a**) and PD-MoS_2-K (**b**) at different reaction temperatures. The product selectivity was calculated on a CO_2-free basis. Catalysts were pretreated in-situ by H_2 at 300 °C for 1 hour before reaction. Reaction activity tests were performed at 50 bar, 3000 mL $g_{cat.}^{-1}$ h^{-1}, and H_2/CO ratio of 2.

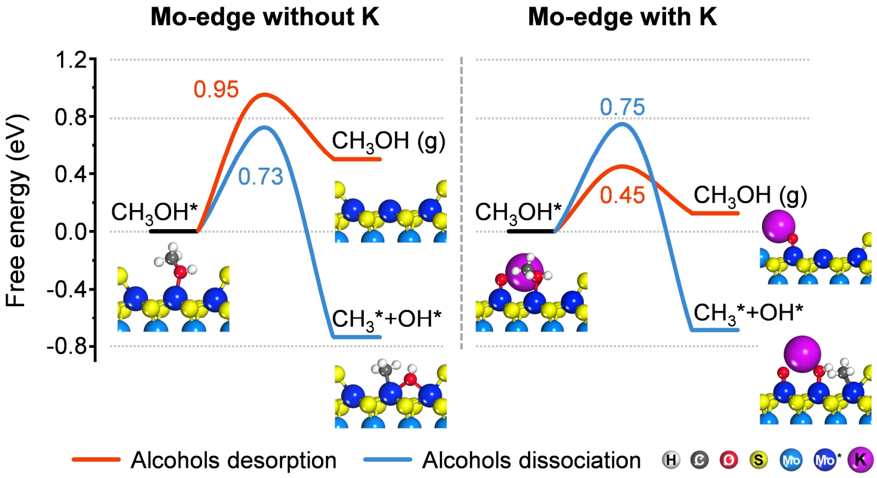

Supplementary Fig. 24. DFT calculations on CH_3OH^* desorption or dissociation on Mo-edge SVs with or without the decoration of potassium.

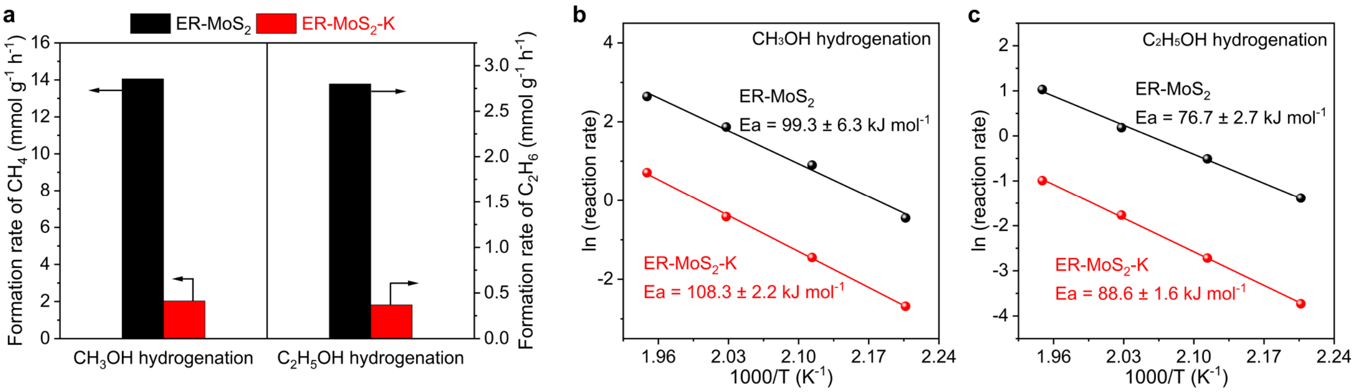

Supplementary Fig. 25. a, Formation rate of CH_4 and C_2H_6 via the hydrogenation of CH_3OH and C_2H_5OH, respectively, over the ER-MoS_2 and ER-MoS_2-K catalysts at 240 °C. **b, c,** Arrhenius plots calculated based on the reaction rate for the hydrogenation of CH_3OH (**b**) and C_2H_5OH (**c**) over ER-MoS_2 and ER-MoS_2-K. Catalysts were pretreated in-situ by H_2 at 300 °C for 1 hour before reaction.

Note: The hydrogenation of both CH_3OH and C_2H_5OH over the ER-MoS_2 catalyst proceed with considerable reaction rates for the formation of CH_4 and C_2H_6, respectively (Supplementary Fig. 25a), suggesting that hydrocarbons can be generated through the hydrogenation of alcohols. With the decoration of potassium, formation rates of CH_4 and C_2H_6 on the ER-MoS_2-K catalyst are significantly suppressed (Supplementary Fig. 25a). However, the apparent activation energies for the hydrogenation of CH_3OH to CH_4 and C_2H_5OH to C_2H_6 over the ER-MoS_2 catalyst are similar to those over the ER-MoS_2-K catalyst (Supplementary Fig. 25b), being consistent with our DFT calculations that potassium modification has little effect on the C-O cleavage activity (Fig. 4e and Supplementary Fig. 24). These results further confirm the effect of potassium in decreasing the competing activation energies of desorption that hinders the C-O cleavage of the CH_3OH^* or $C_2H_5OH^*$.

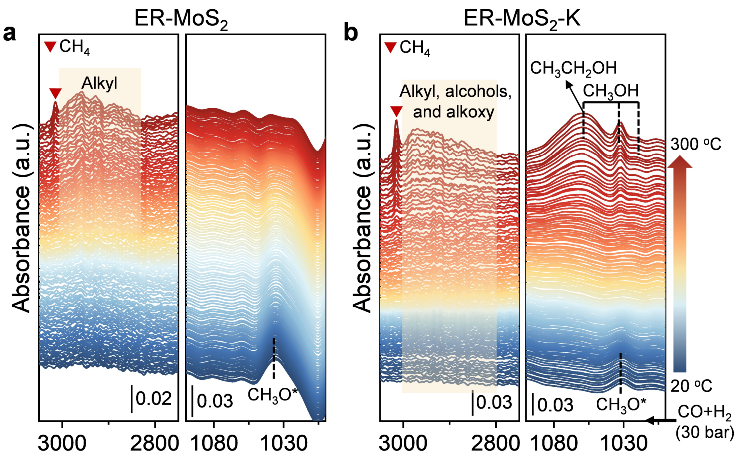

Supplementary Fig. 26. a, b, In-situ DRIFTS of CO hydrogenation over the H_2-pretreated ER-MoS_2 (**a**) and ER-MoS_2-K (**b**) at H_2/CO of 2, 30 bar and different temperatures.

Note: When CO/H_2 mixture passes through the reduced ER-MoS_2 and ER-MoS_2-K at 20 °C, we can observe the appearance of CH_3O* species on both catalysts (Supplementary Figs. 26a, b). With the increase of reaction temperature from 20 to 300 °C, the CH_3O* species on ER-MoS_2 gradually disappear with the rise of gaseous CH_4 peak and band of adsorbed alkyl species such as CH_3* and C_2H_5* (Supplementary Fig. 26a), indicating the transformation of CH_3O* to alkyl species and CH_4 product. It is noteworthy that no signs of HCOO*, CH_3O* species and alcohol products was observed in the DRIFT spectra of ER-MoS_2 at 300 °C, thus the broad band at around 2840~3000 cm^{-1} is attributed to alkyl species on the ER-MoS_2 (Supplementary Fig. 26a). In contrast, obvious increase of vibration bands of CH_3OH and C_2H_5OH was observed on the ER-MoS_2-K at elevated temperatures (Supplementary Fig. 26b). These results show that the decoration of K promotes the formation and desorption of alcohols, thus improving the alcohol selectivity, which is also supported by our DFT calculation (Figs. 4e-g in main text and Supplementary Fig. 24).

Supplementary Table 1. Surface area and pore volume of different MoS_2 samples and SBA-15 template.

Entry	Samples	BET surface area (m^2 g^{-1})	Pore volume (cm^3 g^{-1})	Entry	Samples	BET surface area (m^2 g^{-1})	Pore volume (cm^3 g^{-1})
1	SBA-15	434.6	1.34	2	ER-MoS_2-SBA	391.8	0.99
3	ER-MoS_2	84.3	0.18	4	ER-MoS_2-K	43.6	0.10
5	PD-MoS_2	161.0	0.64	6	PD-MoS_2-K	103.5	0.44
7	ER-MoS_2^{foam}	47.0	0.20	8	ER-MoS_2^{foam}-K	25.4	0.20
9	PD-MoS_2^{foam}	38.5	0.16	10	PD-MoS_2^{foam}-K	29.0	0.16

Note: The SBA-15 template possesses a high surface area of 434.6 m^2 g^{-1} and a high pore volume of 1.34 cm^3 g^{-1} (Entry 1) with a pore diameter of around 10 nm (Supplementary Fig. 1), providing abundant channel space for the confined growth of MoS_2. The ER-MoS_2-SBA as an intermediate sample presents a decreased surface area of 391.8 m^2 g^{-1}, accompanied by the decrease of pore volume to 0.99 cm^3 g^{-1}. After removing the SBA-15 template, the obtained ER-MoS_2 sample possesses a surface area of 84.3 m^2 g^{-1} (Entry 3), which is mainly contributed by MoS_2 edges due to the confined lateral growth of MoS_2 in the channel (Fig. 1c in main text and Supplementary Fig. 2a). In contrast, the template-free synthesized PD-MoS_2 possesses a relatively higher surface area of 161.0 m^2 g^{-1} (Entry 5), which is mainly contributed by MoS_2 basal planes with large lateral sizes (Fig. 1l in main text and Supplementary Fig. 2b). The addition of K promoter decreases the surface area to 43.6 and 103.5 m^2 g^{-1} for the ER-MoS_2-K and PD-MoS_2-K, respectively (Entry 3-6), but without affecting overall pore size distributions and structures (Supplementary Fig. 3). These results illustrate the different morphologies of the ER-MoS_2-K and PD-MoS_2-K catalysts. Therefore, we also synthesized the foam-shaped MoS_2 samples, i.e., the ER-MoS_2^{foam}-K and PD-MoS_2^{foam}-K, possessing similar surface areas and pore sizes (Supplementary Figs. 8a, b, d, e, and Entry 8, 10), to exclude the effect of different morphologies on the selectivity.

Supplementary Table 2. Summary of the element composition of the reduced ER-MoS_2-K and PD-MoS_2-K catalysts from XRF and ICP.

Catalyst	Element composition (XRF, wt %)[a]			Element composition (ICP, wt %)	
	Mo	S	K	Mo	K
ER-MoS_2-K	56.4	38.6	5.0	43.3	3.8
PD-MoS_2-K	56.6	38.4	5.0	41.6	4.3

[a] O element was not included in the calculation because the catalysts had been exposed to air before characterization.

Note: XRF and ICP results show that the two catalysts possess similar composition, indicating the negligible impact of the treatment process with acidic and basic solutions.

Supplementary Table 3. Comparison in $C_{2-4}OH$ selectivity over different catalysts below 300 °C.

Catalyst	Pressure (bar)	Temp. (°C)	$C_{2-4}OH$ sel.[a] (C-mol %)
ER-MoS_2-K (This work)	50	200	38.7
		220	42.2
		240	45.2
PD-MoS_2-K (This work)	50	220	17.9
		240	24.5
		260	22.2
RhMn@S-1[3]	30	290	22.7
Rh-Mn/W_xC[4]	50	300	10.7
RhFe/TiO_2[5]	20	254	34.6
		270	31.0
		294	23.7
K-CuMnAl[6]	40	300	16.0
CuCoAl|*t*-ZrO_2[7]	50	260	27.9
		270	26.6
		280	27.8
CuCo@M-SiO_2[8]	25	260	39.0
CuCoMn[9]	25	270	32.0
CuCo/Al_2O_3/CFs[10]	30	220	43.4
KCuFe/Zeolite[11]	50	270	29.1
CoMn/MAC (P)|Rh/3v-PPh_3@POPs[12]	30	195	16.3[b]
		200	13.8[b]
		220	14.7[b]
CoMn|CuZnAlZr[13]	60	200	6.1[c]
		210	5.3[c]
		220	6.1[c]
		230	9.5[c]
		240	9.5[c]
CoZrLa/AC[14]	30	225	17.3[b]
Fe_2N[15]	20	210	10.9
K-Fe/NC[16]	30	240	16.6[b]
RhK/Mo_2C[17]	30	290	36.5
KMoP/MC[18]	60	270	29.9
K-Co-Mo/AC[19]	50	300	30.0
Ni/K_2CO_3/MoS_2[20]	100	280	29.0
		300	33.4

[a] Calculated on a CO_2-free basis.

[b] $C_{2-5}OH$ selectivity.

[c] Weight selectivity.

Supplementary Table 4. Comparison in $C_{2+}OH$ yield over different MoS_2-based catalysts.

Catalyst	Pressure (bar)	GHSV (mL $g_{cat.}^{-1}$ h^{-1})	Temp. (°C)	$C_{2+}OH$ Yield (C-mol %)
ER-MoS_2-K (This work)	50	1500	240	8.41
		3000	240	5.10
			260	5.65
PD-MoS_2-K (This work)	50	3000	240	2.48
			260	3.69
KCoMoS[21]	103.5	3000 h^{-1}	290	1.32
			330	7.67
			340	9.22
KCoMoS_x[22]	87	4500	360	7.20
K-MoS_2[23]	87	4500	300	3.43
			340	6.10
K,NiMoS/ZnMgAl[24]	50	3000	350	4.22
Mo/K/MMO[25]	103.5	796	310	3.99
		1366		3.25
		4610		1.59
K,Ni-Mo/MMO[26]	50	3200	350	3.56
K,Ni-MoWS_2/MMO[27]	50	2250	350	4.56
K(C)CoMoAl[28]	50	5000 h^{-1}	260	1.85
			280	1.64
			300	2.60
MoKC-MMO[29]	103.5	1497	310	3.48
		2322		2.58
		3936		1.78
		5819		1.36
K-MoS_2/CNT[2]	20	1025	360	0.80

Supplementary Table 5. Catalytic performances of different MoS_2-based catalysts.

Catalyst	CO conv. (%)	CO_2 sel. (%)	Sel.[a] (C-mol %)						
			CH_4	C_xH_y (C_{2+})	CH_3OH	C_2H_5OH	C_3H_7OH	C_4H_9OH	$C_{2\text{-}4}OH$
ER-MoS_2-K	17.0	33.6	18.7	15.4	20.7	31.5	11.1	2.6	45.2
PD-MoS_2-K	12.6	19.2	10.3	7.3	58.2	18.1	5.3	1.1	24.5
ER-MoS_2^{foam}-K	15.0	29.4	18.6	12.8	28.9	27.9	9.5	2.3	39.7
PD-MoS_2^{foam}-K	14.9	24.4	13.8	9.1	47.1	21.5	6.8	1.6	29.9

[a] Calculated on a CO_2-free basis. Tiny amount of DME (sel. < 0.1%) and $C_{5+}OH$ (sel. < 0.3%) could be detected. Catalysts were pretreated in-situ by H_2 at 300 °C for 1 hour before reaction. Reaction activity tests were performed at 50 bar, 240 °C, 3000 mL $g_{cat.}^{-1}$ h^{-1} and H_2/CO ratio of 2.

Supplementary Table 6. DFT-calculated zero-point vibrational energies (E_{ZPE}), enthalpic corrections ($H_{trans+rot+vib}$) and entropic corrections ($TS_{trans+rot+vib}$) at 240 °C for the free molecules.

Molecules	E_{ZPE}	$H_{trans+rot+vib}$	$TS_{trans+rot+vib}$
CO (g)	0.132	0.155	1.014
H_2 (g)	0.267	0.155	0.625
CH_3OH (g)	1.355	0.240	1.427
C_2H_5OH (g)	2.115	0.330	1.676
C_3H_7OH (g)	2.874	0.426	1.925
C_4H_9OH (g)	3.631	0.521	2.165

Supplementary Table 7. Calculated vibrational frequencies in cm^{-1} for the free molecules.

Molecules	Vibrational frequencies (cm^{-1})
CO (g)	2125.139, 29.761, 10.728
H_2 (g)	4305.894, 159.894, 67.117
CH_3OH (g)	3740.974, 3054.394, 2981.522, 2913.801, 1460.237, 1447.208, 1425.706, 1329.243, 1135.603, 1060.620, 1006.805, 297.643, 60.095, 40.546, 29.497
C_2H_5OH (g)	3740.408, 3060.403, 3058.272, 2965.336, 2942.036, 2905.178, 1473.425, 1453.331, 1436.276, 1398.677, 1352.193, 1257.443, 1232.644, 1140.681, 1078.786, 1015.258, 882.929, 804.375, 401.807, 289.035, 236.053, 78.276, 50.357, 12.476
C_3H_7OH (g)	3733.355, 3047.354, 3033.030, 3020.134, 3004.704, 2958.724, 2955.370, 2904.825, 1463.506, 1453.900, 1447.276, 1427.292, 1374.871, 1363.260, 1338.585, 1319.774, 1253.135, 1197.640, 1106.839, 1065.618, 1053.594, 959.506, 885.191, 842.298, 748.840, 464.438, 319.629, 267.604, 227.061, 129.259, 48.570, 40.615, 17.271
C_4H_9OH (g)	3737.408, 3063.872, 3037.529, 3005.069, 2985.494, 2966.066, 2957.254, 2942.634, 2931.284, 2899.325, 1465.832, 1460.652, 1448.769, 1438.928, 1417.697, 1395.776, 1366.999, 1340.032, 1330.897, 1279.520, 1270.300, 1217.131, 1193.709, 1128.084, 1094.160, 1070.511, 999.098, 948.095, 937.969, 839.713, 796.716, 759.351, 467.073, 343.093, 333.913, 238.885, 234.337, 154.939, 79.230, 53.948, 29.092, 18.446

Supplementary Table 8. DFT-calculated zero-point vibrational energies (E_{ZPE}), vibrational internal energy corrections (U_{vib}) and vibrational entropic corrections (TS_{vib}) at 240 °C for the intermediates on the in-plane SVs.

Intermediates	E_{ZPE}	U_{vib}	TS_{vib}
CH_3O* + H*	1.660	0.314	0.596
CH_3OH*	1.412	0.262	0.555
CH_3* + OH*	1.241	0.290	0.533
CH_3* + CO*	1.132	0.281	0.499
CH_3CO*	1.220	0.276	0.545
CH_3* + H*	1.117	0.193	0.343
CH_4*	1.221	0.247	0.653

Supplementary Table 9. Calculated vibrational frequencies in cm^{-1} for the intermediates on the in-plane SVs.

Intermediates	Vibrational frequencies (cm^{-1})
CH_3O^* + H*	2963.898, 2936.122, 2876.022, 1631.548, 1437.421, 1415.294, 1411.126, 1129.829, 1106.704, 1064.274, 925.447, 591.749, 351.141, 289.429, 233.471, 218.363, 193.719, 77.294
CH_3OH^*	3436.896, 3059.887, 3049.047, 2961.249, 1451.659, 1426.530, 1410.137, 1279.824, 1122.763, 1030.423, 956.002, 566.439, 244.463, 226.423, 179.222, 152.623, 126.136, 93.592
CH_3^* + OH*	3632.808, 2912.009, 2781.851, 2536.761, 1345.729, 1275.856, 1231.797, 763.557, 612.232, 503.859, 430.975, 413.025, 361.281, 324.627, 267.765, 258.243, 197.622, 169.725
CH_3^* + CO*	3007.466, 2611.429, 2587.248, 1492.023, 1430.904, 1410.864, 1195.696, 779.302, 571.317, 491.621, 459.413, 413.312, 405.330, 357.973, 325.226, 297.691, 229.297, 200.718
CH_3CO^*	3089.247, 3003.966, 2943.055, 1410.450, 1395.043, 1313.463, 1295.144, 1055.427, 949.831, 918.557, 590.532, 449.106, 324.239, 255.360, 226.721, 190.344, 166.999, 98.242
CH_3^* + H*	3083.860, 3021.895, 2927.213, 1412.280, 1409.707, 1152.798, 1117.909, 742.958, 702.426, 670.305, 608.516, 400.302, 334.985, 296.327, 136.266
CH_4^*	3099.042, 3066.068, 3060.442, 2950.783, 1511.141, 1508.654, 1305.676, 1285.447, 1267.429, 195.292, 145.722, 112.402, 83.621, 77.021, 27.470

Supplementary Table 10. DFT-calculated zero-point vibrational energies (E_{ZPE}), vibrational internal energy corrections (U_{vib}) and vibrational entropic corrections (TS_{vib}) at 240 °C for the intermediates on the Mo-edge SVs.

Intermediates	E_{ZPE}	U_{vib}	TS_{vib}
CH_3O* + H*	1.268	0.270	0.594
CH_3OH*	1.426	0.266	0.594
CH_3* + OH*	1.308	0.286	0.568
CH_3* + CO*	1.171	0.313	0.656
CH_3CO*	1.215	0.290	0.621
CH_3* + H*	1.150	0.197	0.368
CH_4*	1.222	0.225	0.507
C_2H_5* + CO*	1.934	0.410	0.881
C_2H_5CO*	1.973	0.388	0.836
C_2H_5* + H*	1.900	0.302	0.623
C_2H_6*	1.992	0.317	0.762
C_3H_7* + CO*	2.687	0.509	1.100
C_3H_7CO*	2.726	0.492	1.117
C_3H_7* + H*	2.664	0.393	0.796
C_3H_8*	2.753	0.411	0.949

Supplementary Table 11. Calculated vibrational frequencies in cm^{-1} for the intermediates on the Mo-edge SVs.

Intermediates	Vibrational frequencies (cm^{-1})
CH_3O* + H*	3030.634, 3011.894, 2940.944, 1551.595, 1437.629, 1432.924, 1409.388, 1130.876, 1107.067, 1021.261, 668.379, 581.957, 434.957, 233.583, 204.193, 112.036, 90.980, 50.741
CH_3OH*	3547.947, 3105.568, 3076.101, 2986.897, 1451.684, 1445.473, 1421.009, 1310.502, 1138.862, 1057.841, 936.957, 594.898, 277.083, 188.753, 167.959, 134.804, 92.556, 61.589
CH_3* + OH*	3563.442, 3058.903, 3025.935, 2936.524, 1414.496, 1396.710, 1150.042, 824.329, 731.993, 621.729, 535.249, 502.124, 397.944, 270.063, 254.573, 210.544, 126.596, 78.788
CH_3* + CO*	3090.270, 3081.027, 2986.242, 1852.768, 1421.780, 1405.710, 1156.518, 768.550, 718.835, 497.497, 439.316, 407.900, 375.653, 196.438, 166.001, 135.599, 114.059, 74.716
CH_3CO*	3095.431, 3027.031, 2961.051, 1423.027, 1411.613, 1404.865, 1319.648, 1101.215, 958.958, 922.885, 545.927, 396.223, 311.981, 278.154, 166.128, 106.650, 98.588, 74.998
CH_3* + H*	3061.772, 3029.549, 2951.759, 1811.241, 1422.001, 1390.403, 1142.919, 768.859, 707.201, 688.608, 606.534, 430.318, 229.764, 172.910, 144.523
CH_4*	3130.766, 3057.608, 2879.502, 2806.297, 1512.553, 1455.179, 1323.292, 1296.135, 1169.085, 305.839, 229.886, 223.735, 132.226, 109.267, 74.594
C_2H_5* + CO*	3054.152, 3030.289, 3015.024, 2989.541, 2945.326, 1841.940, 1441.093, 1438.501, 1414.984, 1352.128, 1238.048, 1150.103, 1005.982, 964.387, 909.678, 656.989, 490.283, 441.991, 413.741, 358.585, 292.575, 238.324, 176.895, 115.265, 109.495, 63.318, 53.873

$C_2H_5CO^*$	3070.815, 3067.211, 2991.794, 2974.164, 2937.983, 1452.346, 1447.089, 1423.965, 1375.026, 1371.082, 1280.410, 1225.446, 1111.574, 1050.742, 1006.328, 920.694, 726.124, 528.303, 451.175, 361.861, 309.464, 206.180, 187.653, 139.554, 92.550, 64.601, 58.887
$C_2H_5^* + H^*$	3043.145, 3015.993, 2973.837, 2954.852, 2922.117, 1802.153, 1439.605, 1435.834, 1386.673, 1355.848, 1211.576, 1113.434, 1008.222, 928.316, 895.526, 767.272, 690.251, 519.703, 398.901, 259.860, 233.836, 137.401, 96.192, 54.028
$C_2H_6^*$	3066.867, 3061.376, 3025.083, 2989.164, 2853.673, 2741.454, 1469.504, 1450.770, 1445.668, 1429.288, 1370.410, 1297.115, 1174.894, 1133.898, 994.548, 824.592, 776.731, 363.984, 216.172, 141.951, 122.460, 100.104, 54.698, 26.718
$C_3H_7^* + CO^*$	3048.965, 3039.767, 3031.045, 2988.352, 2974.703, 2969.506, 2935.215, 1842.967, 1449.297, 1444.544, 1433.852, 1405.064, 1361.466, 1309.255, 1247.951, 1181.741, 1143.053, 1048.004, 1020.842, 976.391, 874.045, 755.054, 691.725, 494.796, 447.997, 414.975, 379.960, 371.585, 240.783, 195.930, 161.028, 134.862, 118.892, 97.544, 82.475, 24.866
$C_3H_7CO^*$	3054.217, 3043.404, 3019.343, 3005.791, 2974.771, 2970.757, 2919.491, 1456.133, 1451.742, 1447.419, 1440.098, 1379.215, 1367.277, 1334.722, 1289.935, 1256.927, 1193.957, 1096.929, 1047.931, 1029.966, 992.475, 870.015, 833.897, 726.315, 697.697, 402.104, 350.106, 303.884, 248.651, 209.438, 206.773, 131.337, 94.827, 75.959, 35.389, 21.613
$C_3H_7^* + H^*$	3050.021, 3038.664, 2979.295, 2967.950, 2951.073, 2928.802, 2896.361, 1797.358, 1450.921, 1444.910, 1432.443, 1405.356, 1361.769, 1320.322, 1262.920, 1187.630, 1112.731, 1065.319, 987.896, 959.990, 882.546, 799.448, 760.617, 672.532, 561.426, 431.143, 354.556, 273.580, 213.114, 149.317, 135.038, 72.827, 65.335

C_3H_8*	3049.416, 3044.955, 3040.249, 3007.450, 2976.088, 2971.254, 2872.752, 2730.250, 1473.194, 1452.047, 1450.802, 1436.908, 1411.812, 1371.679, 1314.699, 1280.826, 1272.098, 1180.996, 1110.247, 1051.128, 919.539, 882.243, 853.916, 749.162, 370.833, 327.869, 264.133, 173.120, 131.229, 93.581, 63.556, 48.536, 35.125

Supplementary Table 12. Calculated vibrational frequencies in cm^{-1} for the reacting species at the transition states on the in-plane SVs. The frequencies in the brackets denote the imaginary frequencies of the transition states.

Reaction	Vibrational frequencies (cm^{-1})
$CH_3O^* + H^* \rightarrow CH_3OH^*$	2983.533, 2939.857, 2852.685, 1441.375, 1423.826, 1415.604, 1196.427, 1117.900, 1113.147, 1036.144, 657.144, 529.402, 338.844, 253.645, 188.333, 175.592, 87.594, [346.904]
$CH_3OH^* \rightarrow CH_3^* + OH^*$	3647.842, 3220.636, 3200.231, 3029.149, 1373.429, 1348.906, 809.729, 735.004, 485.251, 444.866, 425.933, 342.588, 266.738, 156.486, 121.824, 96.190, 65.389, [255.289]
$CH_3^* + CO^* \rightarrow CH_3CO^*$	3115.128, 3057.575, 2969.191, 1627.744, 1396.180, 1343.326, 1193.808, 831.558, 818.477, 496.088, 423.789, 311.000, 267.647, 249.705, 208.428, 143.439, 105.964, [318.541]
$CH_3^* + H^* \rightarrow CH_4^*$	3074.689, 3034.401, 2941.357, 1564.617, 1416.532, 1407.260, 1157.501, 876.661, 810.976, 517.407, 400.831, 320.087, 294.684, 140.474, [772.950]

Supplementary Table 13. Calculated vibrational frequencies in cm^{-1} for the reacting species at the transition states on the Mo-edge SVs. The frequencies in the brackets denote the imaginary frequencies of the transition states.

Reaction	Vibrational frequencies (cm^{-1})
CH_3O^* + H^* → CH_3OH^*	3049.843, 3042.053, 2959.459, 1443.615, 1433.791, 1410.620, 1148.974, 1130.797, 1064.237, 976.786, 930.997, 371.383, 219.025, 191.948, 115.432, 76.351, 53.255, [1260.816]
CH_3OH^* → CH_3^* + OH^*	3605.506, 3219.860, 3203.382, 3036.329, 1386.140, 1372.480, 936.004, 802.125, 669.378, 618.252, 612.649, 376.867, 203.448, 142.490, 130.366, 84.960, 33.159, [401.502]
CH_3^* + CO^* → CH_3CO^*	3091.167, 3018.527, 2890.783, 1788.861, 1405.532, 1362.392, 1267.193, 1013.563, 860.207, 578.577, 514.021, 372.859, 304.466, 284.468, 216.963, 140.084, 80.844, [46.428]
CH_3^* + H^* → CH_4^*	3061.900, 3025.750, 2952.786, 1909.819, 1425.560, 1396.012, 1210.371, 938.377, 871.045, 447.183, 421.180, 147.726, 90.383, 66.540, [857.330]
$C_2H_5^*$ + CO^* → $C_2H_5CO^*$	3047.486, 3041.240, 3022.551, 2983.094, 2961.472, 1591.406, 1455.258, 1443.384, 1381.635, 1363.313, 1229.693, 1218.489, 1068.961, 986.256, 982.161, 752.212, 702.678, 490.525, 407.195, 275.480, 214.119, 166.501, 133.157, 86.822, 69.545, 5.709, [122.602]
$C_2H_5^*$ + H^* → $C_2H_6^*$	3078.077, 3041.172, 3008.311, 2954.284, 2933.429, 1967.701, 1441.295, 1434.655, 1383.335, 1353.710, 1272.602, 1209.060, 1014.839, 1004.900, 934.111, 728.180, 626.987, 344.339, 237.983, 210.356, 91.670, 71.780, 10.765, [825.583]

$C_3H_7^* + CO^* \rightarrow C_3H_7CO^*$	3049.409, 3046.639, 3017.804, 3002.925, 2975.943, 2971.021, 2768.539, 1798.697, 1451.631, 1448.346, 1435.463, 1376.105, 1364.738, 1314.520, 1274.908, 1236.222, 1184.287, 1067.993, 1055.942, 1025.188, 895.408, 860.782, 745.803, 643.075, 533.842, 351.873, 337.276, 273.168, 259.385, 192.331, 122.166, 91.804, 56.686, 34.198, 24.219, [69.414]
$C_3H_7^* + H^* \rightarrow C_3H_8^*$	3050.267, 3035.143, 2999.205, 2980.182, 2966.867, 2944.256, 2910.788, 1958.048, 1448.489, 1442.937, 1432.886, 1386.628, 1363.783, 1321.398, 1265.582, 1230.083, 1194.121, 1063.182, 1038.528, 1025.551, 854.081, 793.242, 725.364, 651.561, 362.931, 332.852, 255.375, 211.544, 139.335, 114.397, 66.730, 47.656, [860.433]

Supplementary References

1 Santos, V. P. *et al.* Mechanistic insight into the synthesis of higher alcohols from syngas: The role of K promotion on MoS_2 catalysts. *ACS Catal.* **3**, 1634-1637 (2013).

2 Liu, C., Virginie, M., Griboval-Constant, A. & Khodakov, A. Y. Potassium promotion effects in carbon nanotube supported molybdenum sulfide catalysts for carbon monoxide hydrogenation. *Catal. Today* **261**, 137-145 (2016).

3 Wang, C. *et al.* Direct conversion of syngas to ethanol within zeolite crystals. *Chem* **6**, 646-657 (2020).

4 Won, B. D. *et al.* Rh-Mn/tungsten carbides for direct synthesis of mixed alcohols from syngas: Effects of tungsten carbide phases. *Microporous Mesoporous Mater.* **255**, 44-52 (2018).

5 Haider, M., Gogate, M. & Davis, R. Fe-promotion of supported Rh catalysts for direct conversion of syngas to ethanol. *J. Catal.* **261**, 9-16 (2009).

6 Liakakou, E. T., Isaacs, M. A., Wilson, K., Lee, A. F. & Heracleous, E. On the Mn promoted synthesis of higher alcohols over Cu derived ternary catalysts. *Catal. Sci. Technol.* **7**, 988-999 (2017).

7 Huang, C. *et al.* Direct conversion of syngas to higher alcohols over a CuCoAl|t-ZrO_2 multifunctional catalyst. *ChemCatChem* **13**, 3184-3197 (2021).

8 Chen, G. *et al.* In Situ encapsulated CuCo@M-SiO_2 for higher alcohol synthesis from biomass-derived syngas. *ACS Sustainable Chem. Eng.* **9**, 5910-5923 (2021).

9 Sun, K. *et al.* Design and synthesis of spherical-platelike ternary copper-cobalt-manganese catalysts for direct conversion of syngas to ethanol and higher alcohols. *J. Catal.* **378**, 1-16 (2019).

10 Wang, L., Cao, A., Liu, G., Zhang, L. & Liu, Y. Bimetallic CuCo nanoparticles derived from hydrotalcite supported on carbon fibers for higher alcohols synthesis from syngas. *Appl. Surf. Sci.* **360**, 77-85 (2016).

11 Luk, H. T. *et al.* Impact of carrier acidity on the conversion of syngas to higher alcohols over zeolite-supported copper-iron catalysts. *J. Catal.* **371**, 116-125 (2019).

12 Li, Y. *et al.* Highly selective conversion of syngas to higher oxygenates over tandem catalysts. *ACS Catal.* **11**, 14791-14802 (2021).

13 Lin, T. *et al.* Direct production of higher oxygenates by syngas conversion over a multifunctional catalyst. *Angew. Chem. Int. Ed.* **58**, 4627-4631 (2019).

14 Lebarbier, V. M. *et al.* Effects of La_2O_3 on the mixed higher alcohols synthesis from syngas over Co catalysts: A combined theoretical and experimental study. *J. Phys. Chem. C* **115**, 17440-17451 (2011).

15 Li, L. *et al.* Efficient one-pot synthesis of higher alcohols from syngas catalyzed by iron nitrides. *ChemCatChem* **12**, 1939-1943 (2020).

16 Chen, Y. *et al.* Carbon-supported Fe catalysts with well-defined active sites for highly selective alcohol production from Fischer-Tropsch synthesis. *Appl. Catal. B Environ.* **312**, 121393 (2022).

17 Wang, C. *et al.* Direct synthesis of higher alcohols from syngas over modified Mo_2C catalysts under mild reaction conditions. *Catal. Sci. Technol.* **12**, 1697-1708 (2022).

18 ten Have, I. C. *et al.* Development of molybdenum phosphide catalysts for higher alcohol synthesis from syngas by exploiting support and promoter effects. *Energy Technol.* **7**, 1801102 (2019).

19 Lv, M. *et al.* Activated-carbon-supported K-Co-Mo catalysts for synthesis of higher alcohols from syngas. *Catal. Sci. Technol.* **5**, 2925-2934 (2015).

20 Li, D. *et al.* The performances of higher alcohol synthesis over nickel modified K_2CO_3/MoS_2 catalyst. *Fuel Process. Technol.* **88**, 125-127 (2007).

21 Simeonov, K. *et al.* CoMoS/K catalysts for higher alcohol synthesis from syngas prepared by mechano-chemical activation of molybdenite. *Catal. Sci. Technol.* **4**, 922-924 (2014).

22 Xi, X. *et al.* Enhanced C3+ alcohol synthesis from syngas using KCoMoSx catalysts: effect of the Co-Mo ratio on catalyst performance. *Appl. Catal. B Environ.* **272**, 118950 (2020).

23 Zeng, F. *et al.* Synthesis of mixed alcohols with enhanced C3+ alcohol production by CO hydrogenation over potassium promoted molybdenum sulfide. *Appl. Catal. B Environ.* **246**, 232-241 (2019).

24 Zhang, X. *et al.* Enhanced higher alcohol synthesis from CO hydrogenation on Zn-modified MgAl-mixed oxide supported KNiMoS-based catalysts. *Ind. Eng. Chem. Res.* **59**, 1413-1421 (2020).

25 Morrill, M. R. *et al.* Origins of unusual alcohol selectivities over mixed MgAl oxide-supported K/MoS_2 catalysts for higher alcohol synthesis from syngas. *ACS Catal.* **3**, 1665-1675 (2013).

26 Yong, J. *et al.* Tuning the metal-support interaction in supported K-promoted NiMo catalysts for enhanced selectivity and productivity towards higher alcohols in CO hydrogenation. *Catal. Sci. Technol.* **7**, 4206-4215 (2017).

27 Luan, X. *et al.* Tungsten-doped molybdenum sulfide with dominant double-layer structure on mixed MgAl oxide for higher alcohol synthesis in CO hydrogenation. *Ind. Eng. Chem. Res.* **57**, 10170-10179 (2018).

28 Toyoda, T., Minami, T. & Qian, E. W. Mixed alcohol synthesis over sulfided molybdenum-based catalysts. *Energy Fuels* **27**, 3769-3777 (2013).

29 Taborga Claure, M. *et al.* Tuning of higher alcohol selectivity and productivity in CO hydrogenation reactions over K/MoS_2 domains supported on mesoporous activated carbon and mixed MgAl oxide. *J. Catal.* **324**, 88-97 (2015).
